# Molecular basis for the behavioral effects of the odorant degrading enzyme Esterase 6 in *Drosophila*

**DOI:** 10.1038/srep46188

**Published:** 2017-04-10

**Authors:** Faisal Younus, Nicholas J. Fraser, Chris W. Coppin, Jian-Wei Liu, Galen J. Correy, Thomas Chertemps, Gunjan Pandey, Martine Maïbèche, Colin J. Jackson, John G. Oakeshott

**Affiliations:** 1 CSIRO Land and Water, Black Mountain, Canberra, ACT, 2601, Australia; 2 Research School of Chemistry, Australian National University, Canberra, ACT, 2601, Australia; 3 Université Pierre et Marie Curie, Institut d’Ecologie et des Sciences de l’Environnement de Paris, 75252, Paris, France

## Abstract

Previous electrophysiological and behavioural studies implicate esterase 6 in the processing of the pheromone cis-vaccenyl acetate and various food odorants that affect aggregation and reproductive behaviours. Here we show esterase 6 has relatively high activity against many of the short-mid chain food esters, but negligible activity against cis-vaccenyl acetate. The crystal structure of esterase 6 confirms its substrate-binding site can accommodate many short-mid chain food esters but not cis-vaccenyl acetate. Immunohistochemical assays show esterase 6 is expressed in non-neuronal cells in the third antennal segment that could be accessory or epidermal cells surrounding numerous olfactory sensilla, including basiconics involved in food odorant detection. Esterase 6 is also produced in trichoid sensilla, but not in the same cell types as the cis-vaccenyl acetate binding protein LUSH. Our data support a model in which esterase 6 acts as a direct odorant degrading enzyme for many bioactive food esters, but not cis-vaccenyl acetate.

Insects’ olfactory systems are both primary drivers of their interactions with the environment and an emerging model for studying the molecular basis of eukaryote signaling processes. They are also of enormous interest in applied entomology because they are the targets for various pest control strategies based on mating disruption^1^. Many aspects of insects’ olfactory system have recently been elucidated but others, such as their odorant degrading enzymes (ODEs), are still poorly understood^2^,^3^. It is proposed that ODEs are vital in the maintenance of the ongoing sensitivity of the olfactory system to incoming signals through the rapid inactivation of the relevant pheromones and kairomones once they have activated their receptors^2^,^4^. However few of these have yet been characterized in any detail and fundamental questions remain about their modes of action. In particular there is ongoing debate, both about whether individual ODEs are specific for particular odorants or act generally against many^2^, and about whether they act alone or in combination with odorant binding proteins (OBPs)^2^,^5^. OBPs have been strongly implicated in the transport of incoming odorants through the sensillar lymph to their corresponding receptors, but any subsequent role for them in the deactivation process remains controversial^2^.

Most of the work to date on ODEs has been done on certain Lepidoptera that have antennae large enough for classical biochemical and physiological studies^4^. One of the best characterized is the antennal specific esterase Apo1SE from the giant silk moth *Antheraea polyphemus*, which is estimated to have a *k*_cat_ of 127 s^−1^ for its natural E6Z11-16:acetate pheromone substrate^6^, but little activity for other isomers of this compound or for several other volatile esters tested. Relatively high *k*_cat_ values for their putative pheromone ester substrates have also been reported for a few other lepidopteran antennal esterases, although in at least two of these cases their substrate ranges seem be to less specific^3^,^7^,^8^, perhaps suggesting broad rather than specific ODE functions.

By far the best characterized ODE for the model insect *Drosophila melanogaster* is esterase 6 (EST6). This enzyme was originally reported to degrade the major volatile sex and aggregation pheromone cis-vaccenyl acetate (cVA)^9^. Subsequent electrophysiological comparisons of EST6 wildtype and null flies on comparable genetic backgrounds have confirmed a role for the enzyme in the dynamics of cVA processing^10^. A specific OBP, LUSH, has been identified for cVA in *D. melanogaster* but the latest genetic evidence suggests that the interaction of cVA with its receptor OR67d is independent of LUSH^11^. Notably, the distribution of EST6 in the third antennal segment also includes sensilla that are responsive to other odorants but not to cVA^10^, and further biochemical, electrophysiological and behavioral comparisons of the EST6 active and null strains indicate that the enzyme also acts on various short chain fatty acid food esters^12^,^13^. There is indeed some relationship between the level of EST6 activity for the different esters and the size of the electrophysiological effect^13^, suggesting that EST6 does act as a general ODE with activities for several ester odorants.

As further evidence for pleiotropic effects of the enzyme, EST6 is also known to be expressed at high levels in the male ejaculatory duct, from where it is transferred to the female reproductive tract during mating^14^. It is then rapidly (within minutes) translocated to her hemolymph, where it remains for several days. Comparisons of females mated with null and wildtype EST6 males indicate it acts in the female to stimulate her egg-laying and delay her receptivity to re-mating^15^,^16^. Early claims that this effect was mediated by EST6 action on endogenous cVA^9^ have since been refuted^17^, but the substrate responsible for the effect nevertheless remains unknown.

EST6 is a member of the carboxyl/cholinesterase (CCE) family of proteins^18^, which is represented by 30–110 different gene/enzyme systems encoding diverse functions in the insect genomes so far sequenced^19^. However, the juvenile hormone esterase from the moth *Manduca sexta (Ms*JHE)^20^, an insecticide metabolizing carboxylesterase from the blowfly *Lucilia cuprina (Lc*αE7)^21^ and acetylcholinesterase from *D. melanogaster (Dm*AChE)^22^ are the only insect CCEs for which crystal structures have been determined, so relatively little is known of the structure-function relationships underlying their diverse functions. The structural features of EST6 have so far been inferred from the structure of the *D. melanogaster* AChE or its orthologue from the electric ray *Torpedo californica*, but the low sequence similarity between EST6 and AChE (27%) means that the fine structural features of the enzyme responsible for its substrate specificity have not yet been understood^23^.

In this paper, we present a comprehensive analysis of the substrate range of semi-purified EST6, showing it has significant activity for a range of short chain fatty acid esters but negligible activity for long chain fatty acid esters. In particular, we find that EST6 is not active against cVA, either in the presence or absence of LUSH, but does degrade various volatiles emitted by rotting fruits and the yeasts therein on which the flies naturally live; these volatiles have recently been shown to be key regulators of *Drosophila* mating behavior^24^. We also present a crystal structure for the enzyme which, together with *in silico* docking studies, supports the kinetic data and shows that its active site can readily accommodate short chain fatty acid esters, including the yeast and fruit volatiles above, but not long chain fatty acid esters like cVA. A unique active site location and entry is identified, which appears to explain the enzyme’s substrate preferences. Finally, we present data from immunohistochemical and behavioral assays with RNAi knock-down constructs that localize the expression of EST6 to a large proportion of non-neuronal cells surrounding the olfactory neurons of almost all the olfactory sensilla, but in different cells than those producing LUSH in the trichoid sensilla.

## Results

### Enzyme kinetics

**Figure 1:**
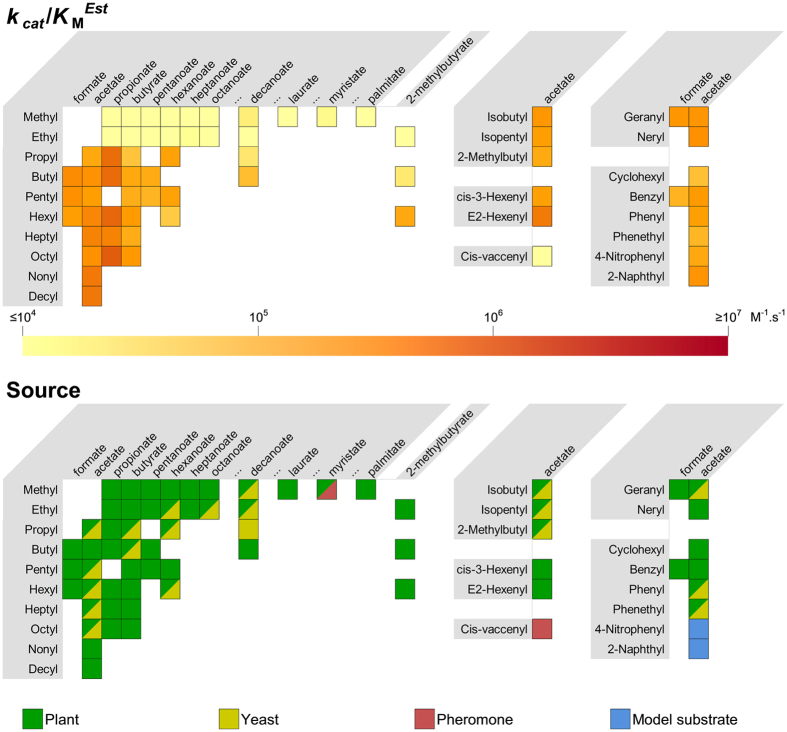
EST6 *k*_cat_/*K*_M_^Est^ and biological source of the most active substrates tested and other substrates of particular structural or physiological significance. Alcohol moieties are listed on the vertical and are grouped according to structural similarity. Acid moieties are listed on the horizontal. An ellipsis (…) demarcates a break in an otherwise incremental series. Data on the biological source of the substrates are taken from Supplementary Table S2. Activity results for all 87 compounds tested are given in Supplementary Table S1.

Wildtype EST6 was tested for activity against 85 bioactive ester odorants and two model substrates; 4-nitrophenyl acetate (4 NPA) and 2-naphthyl acetate (2 NA). It showed detectable activity (generally, a specificity constant *k*_cat_/*K*_M_^Est^ > 1.5 × 10^4^ M^−1^.s^−1^) for 47 of the bioactive esters as well as the two model substrates (Fig. 1 and Supplementary Table S1). Specificity constants for most (42) of these 49 were above 1 × 10^5^ M^−1^.s^−1^, although none exceeded 1.3 × 10^6^ M^−1^.s^−1^, consistent with typical *k*_cat_/*K*_M_ values for enzymatic reactions in secondary metabolism^25^. The highest activities were seen with esters containing longer (C > 6) or more complex (branched, unsaturated or cyclic) leaving groups and acetate or propionate acid moieties, although a combination of mid-length leaving groups and acid groups (butyl decanoate) was also a relatively good substrate in these assays. The 38 compounds for which little or no activity could be detected were mainly methyl or ethyl esters or those with more complex acidic groups. cVA, which has a very long leaving group, was not hydrolysed at significant rates.

Precise *K*_M_ values for most substrates could not be calculated because of low substrate solubility. However, estimates of *K*_M_ values could be obtained for some of the more soluble esters (4 NPA, 2 NA, benzyl acetate, phenyl acetate, phenethyl acetate) and were found to be in the range 121–880 μM under these assay conditions, which included 5% ethanol (Supplementary Table S1). Previous kinetic analyses of EST6 with 2NA^26^ and 4 NPA (Supplementary Fig. S1) indicated the *K*_M_ values were ~5–20 fold lower in the absence of 5% ethanol. The *K*_M_ values that were obtained generally exceed the concentration of substrate in the reaction mixtures (200 μM), which means that the *k*_cat_/*K*_M_^Est^ calculated will be a reasonable approximation of the true *k*_cat_/*K*_M_ value (in those cases where *K*_M_ is lower than 200 μM, the estimated value will underestimate the true *k*_cat_/*K*_M_ for the assay conditions used – see Methods). Given the measured *K*_M_ values are typically >100 μM, the measured *k*_cat_/*K*_M_ values therefore imply relatively high *k*_cat_ values (in some cases >1,000 s^−1^). These results indicate that EST6 is a relatively “fast” enzyme (high *k*_cat_ values) that displays broad specificity, working moderately efficiently with a very wide range of natural esters. In comparison, the related enzyme acetylcholinesterase catalyses acetylcholine hydrolysis with very high efficiency but has an extraordinarily narrow substrate range, essentially catalysing a single substrate^18^.

The assays with cVA were repeated in the presence of the cVA binding protein LUSH, which again indicated negligible activity, even in the presence of a great excess of EST6 (57 nM compared with the 3 nM used previously). The only other known pheromone among the compounds tested was the fatty acid ester methyl myristate, which is also a plant volatile and functions as an attractant to *D. melanogaster*^27^. EST6 also had relatively little activity with this compound (~1.5 × 10^4^ M^−1^.s^−1^).

Apart from the two pheromones and two model substrates, all the esters tested for which EST6 was found to have significant activity are food odorants that are known to be bioactive against *D. melanogaster* in *in vivo* (behavioral) and/or *in vitro* (receptor binding) assays (Supplementary Table S2)^27^,^28^,^29^. Five of the major odorant receptors in this species that are known to have affinity for ester ligands (Or10a, Or22a, Or35a, Or67a and Or98a) all bind a variety of such esters, with substantially overlapping ranges^30^,^31^. Notably, many of the alcohol and aldehyde metabolites of these esters are also known ligands for various *D. melanogaster* odorant receptors^32^.

### Structure determination of EST6

One of the main barriers to crystallizing EST6 was its very low soluble expression in *Escherichia coli*. To address this, we used the same approach as we did to solve the structures of the α-Esterase 7 carboxylesterase from *Lucilia cuprina*^21^. Briefly, we utilized directed evolution to screen libraries of EST6 variants lacking the N-terminal signal peptide^33^ for enhanced activity (as a result of enhanced soluble expression) in *E. coli* (Supplementary Fig. S2). After six rounds of directed evolution, the EST6 variant with greatest soluble expression (EST6-1) contained 16 mutations; K15V, V145L, R208K, G229E, N237S, T247A, D290G, I292F, I335V, E383G, S400G, A416V, F450S, F456S, N485D, I511T (note that amino acids are numbered from the first residue of the mature EST6 protein as it would be processed in its native form within the fly^33^ and omits the start methionine included to permit heterologous expression in *E. coli*). Four of these mutations have been found in EST6 from several *Drosophila* species (V145A, R208K, T247A and I292F)^23^,^34^. Importantly, the catalytic activity of EST6-1 was very similar to that of EST6-WT (Supplementary Fig. S3), suggesting that the 16 mutations principally affected folding, rather than function, consistent with their being located remote from the active site.

Using the Origami B strain of *E. coli*, a cell line that has been designed to enhance disulfide bond formation in the cytoplasm in prokaryotic systems^35^, high levels of soluble EST6-1 were expressed (~20 mg. l^−1^) (Supplementary Fig. S4). Expression of EST6-WT in *E. coli* Origami B cells resulted in substantially lower soluble expression (~0.5 mg. l^−1^). Size exclusion chromatography showed EST6-1 eluted primarily as a monomer, although there was secondary peak present that indicted a small amount of dimer (Supplementary Fig. S4). Crystallization trials of the EST6-1 monomer fraction at two different concentrations did not yield crystals. We then performed surface lysine methylation, which has been shown to increase the propensity of proteins to crystallize^36^, which yielded crystals in conditions of 0.2 M ammonium acetate, 0.1 M Tris pH 8.5 and 25% *w/v* PEG 3,350 that diffracted to 2.1 Å resolution.

**Figure 2:**
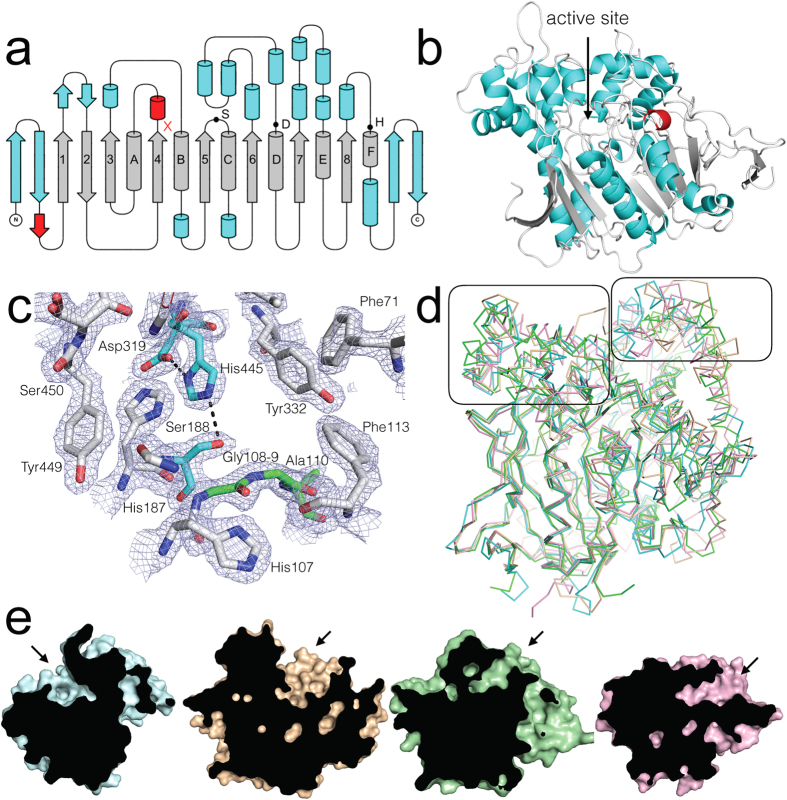
The structure of EST6 from *D. melanogaster*. (**a**) Topology representation of EST6 displaying the conserved α/β-hydrolase fold (grey), secondary structure found in the structurally similar proteins (blue) and unique secondary structure (red). S, D, H represent the Ser188, Asp319 and His445 residues that make up the catalytic triad. The oxyanion hole is located in the loop following sheet 4 (marked by a red x). (**b**) Cartoon diagram of EST6 with features shown in the topology model similarly coloured. The location of the active site is indicated. (**c**) The active site of EST6 with 2*mF*_o_−*dF*_c_ electron density contoured at 1.5 σ. The active site serine and histidine from the catalytic triad are coloured cyan, the oxyanion hole (Gly108, Gly109, Ala110) is coloured green. (**d**) An overlay of EST6 (cyan), *Lc*αE7 (tan; 4FNM), *Dm*AChE (green; 1QO9) and *Ms*JHE (pink; 2FJ0). Conservation of the core β-sheet and conserved α-helices is apparent, but the structures diverge in the region that forms the active site entrance. These regions, either side of the active site, are boxed for clarity. (**e**) A superposition of EST6, LcαE7, DmAChE and MsJHE, with cut-aways through the middle of the active site. The location of the active site entrance difference between EST6 (cyan) and the other related insect carboxylesterases *Lc*αE7 (tan; 4FNM), *Dm*AChE (green; 1QO9) and *Ms*JHE (pink; 2FJ0).

The structure of EST6-1 contains 520 amino acids, 353 water molecules, 32 surface carboxylated lysines and one monomer per asymmetric unit. All but the first four N-terminal amino acids are present in reasonable electron density. EST6-1 adopts an α/β-hydrolase fold, including the conserved catalytic triad and oxyanion hole (Fig. 2a,b). The eight-stranded β-sheet (β1–8) surrounded by six α-helices (A-F), that comprises the canonical fold is present, along with the two antiparallel β-strands at the start and two antiparallel β-strands at the end of the structure that are found in the other three insect carboxylesterases whose structures have been solved^20^,^21^,^22^. The entrance of the active site is formed by loops following β1, loops and two helices following β4, and loops following β8, including helix F that makes up part of the canonical α/β hydrolase fold. The active site itself is formed from the catalytic triad (Ser188, His445 and Asp319), oxyanion hole (Gly108, Gly109 and Ala189) and additional residues (Tyr322 Tyr449, Phe450, Asn455, Phe456 and Val457) from a helix after β7 and a loop following β5 (Fig. 2c). There are three intramolecular disulfide bonds present (65–84, 240–252, and 493–514) on surface loop regions. The first two disulfide bonds are also seen in the other two insect carboxylesterase structures containing disulfides (AChE, JHE), but the third disulfide is unique to EST6, which also has a shortened C-terminus relative to the other three carboxylesterases structures.

### Comparison to known structures

Analysis of the ESTHER database^37^, which comprehensively describes the α/β hydrolase fold across a wide range of organisms, reveals that EST6-1 falls into Block C, which also includes the other three known insect carboxylesterase structures. Amongst the insect carboxylesterases, which Oakeshott *et al*.^18^ have divided into 14 Clades, EST6-1 falls into Clade E, with *Lc*αE7 (PDB - 4FNM) in Clade B, *Ms*JHE (PDB - 2FJO) in Clade G and *Dm*AChE (PDB - 1QO9) in Clade J. Application of the SALAMI server^38^ confirmed there were no structural homologues in PDB closer to EST6-1 than these three enzymes (Supplementary Table S3). The four clades are well separated from one another phylogenetically (26–29% amino acid identity) but all four structures superimpose well over the canonical fold (2.27, 2.09 and 2.41 Å C-α r.m.s.d. for the other three compared with EST6-1, respectively). In contrast, in the loop regions above the canonical β-sheet and α-helices, there is significant variance between the structures (Fig. 2d).

Closer inspection of the structures and alignment revealed that EST6-1 is missing the C-terminal helix present in *Dm*AChE, *Lc*αE7 and *Ms*JHE. Another feature of interest is the length and composition of the surface-exposed loop regions after strands β1, β6 and β8, which contribute to the active site entrance in the other three proteins. In the latter three, the opening of the active site is formed from helices after β6 and β7 and loops and helices after β1 and β8, but this region has closed over in EST6-1. Its active site entry is instead formed by loops and helices after β1, β4, and β8 on the opposite face of the protein (Fig. 2e). The result is a narrower and shorter active site entrance in EST6-1 in comparison to the open and accessible active site in *Lc*αE7 and the deep gorges leading to the catalytic triads in AChE and JHE.

A comparison of the four structures using the CASTp server^39^ also revealed that the active site volume of EST6-1 was significantly less than in *Lc*αE7 (Table 1). The relative sizes of the active sites of *Dm*AChE, *Lc*αE7 and *Ms*JHE reflect their native substrate preferences: *Lc*αE7 natively hydrolyses a wide range of medium chain fatty acid methyl esters and has a large active volume (2727 Å^3^)^21^, while AChE and JHE both have narrower substrate specificities, for the smaller acetylcholine and juvenile hormone molecules respectively, and have much smaller active site volumes, of 782 and 1308 Å^3^, respectively. The active site volume of EST6-1 is estimated to be 935 Å^3^, which is consistent with the observed preference of EST6 for smaller substrates than *Lc*αE7 (Fig. 1).

### The substrate binding pocket

**Figure 3:**
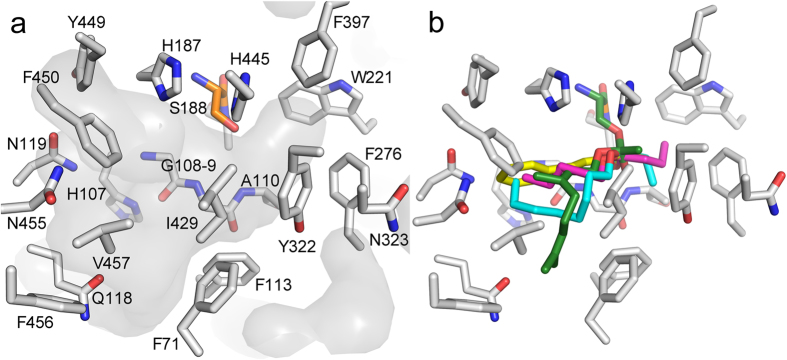
The substrate binding site of EST6. (**a**) The surface of the substrate binding site is shaded grey and the residues that comprise the small and large pockets are shown (grey) as is the catalytic serine (orange). The small site consists of Ala110, Trp221, Phe276, Tyr322, Phe397 and His445, and the large site consists of Gln70, Phe71, Phe113, Gly114, Gln118, Asn119, Ile429, Tyr449, Phe450, Asn455, Phe456, and Val457. (**b**) An overlay of representative acylated enzyme intermediates covalently docked into EST6: the efficiently hydrolyzed substrates pentyl butyrate (magenta), octyl propionate (cyan), geranyl acetate (green) and phenethyl acetate (yellow) all produce acylated intermediates that are accommodated by the substrate binding site.

Given that EST6-1 is ~97% identical to EST6-WT, and the mutations distinguishing them are all remote from the active site, it is highly likely that the structures will be essentially identical in this region. Nevertheless, for analysis of the substrate binding site, a model of EST6-WT was produced using the empirical structure of EST6-1 and the FoldX force field, which has been developed to allow accurate modeling of point mutations, among other things^40^. As noted above, the conserved catalytic triad of EST6 consists of Ser188, His445 and Asp319, while the backbone NH groups of Gly108, Gly109 and Ala189 create the oxyanion hole (Fig. 2c). His187 is adjacent to the catalytic serine and as with the other three structures its side chain extends into the active site; in the others it has been suggested to affect substrate specificity^26^. EST6 has an asymmetrical binding pocket with a very small, hydrophobic and buried sub-site consisting of Ala110, Trp221, Phe276, Tyr322, Phe397 and His445 that could accommodate the carboxyl group. Opposite this, there is a larger cavity (the putative alcohol leaving group site) that extends into the active site exit/entrance and is slightly less hydrophobic, consisting of Gln70, Phe71, Phe113, Gly114, Gln118, Asn119, Ile429, Tyr449, Phe450, Asn455, Phe456 and Val457 (Fig. 3a).

A representative range of potential substrates that EST6 was tested with were docked into the active site of EST6 using flexible docking with DOCKovelent^41^, which is able to screen binding modes for substrates or inhibitors that form covalent bonds with the target enzyme (Fig. 3b, Supplementary Fig. S5 and Supplementary Table S4). The docking results are entirely consistent with the kinetic data, in so much as acylated enzyme intermediates for substrates that were hydrolyzed at significant rates were well accommodated by the substrate binding pocket, whereas no suitable binding poses (without steric clashes) could be obtained for the acylated enzyme intermediates that would result from reaction with compounds that were shown not to be substrates of EST6 (such as cVA). A clear trend is evident: the small sub-site can easily accommodate chains of 1–6 carbons, while the leaving group site has a preference for longer saturated chains, such as hexyl and octyl, over smaller chains, such as methyl and ethyl, but not as large as cVA (C18). This is also consistent with the high activity and complementary binding of geranyl and neryl acetate, with the short carboxyl side chains being accommodated in the small sub-site and the unsaturated leaving group being accommodated in the leaving group site. Likewise, those substrates with aromatic leaving groups and short carboxyl groups are also well accommodated (Supplementary Fig. S5). This analysis provides a molecular explanation for the observed substrate preference for typical food odorants with carboxyl groups of 0–6 carbons and leaving alcohol groups up to ~10 carbons, including branched and aromatic moieties. This structural analysis also strongly supports the kinetic analysis and the initially surprising observation that cVA does not appear to be a physiological substrate for EST6, in that it is clearly far too large for the EST6 substrate binding pocket.

### Localization of EST6 in the antennae

**Figure 4:**
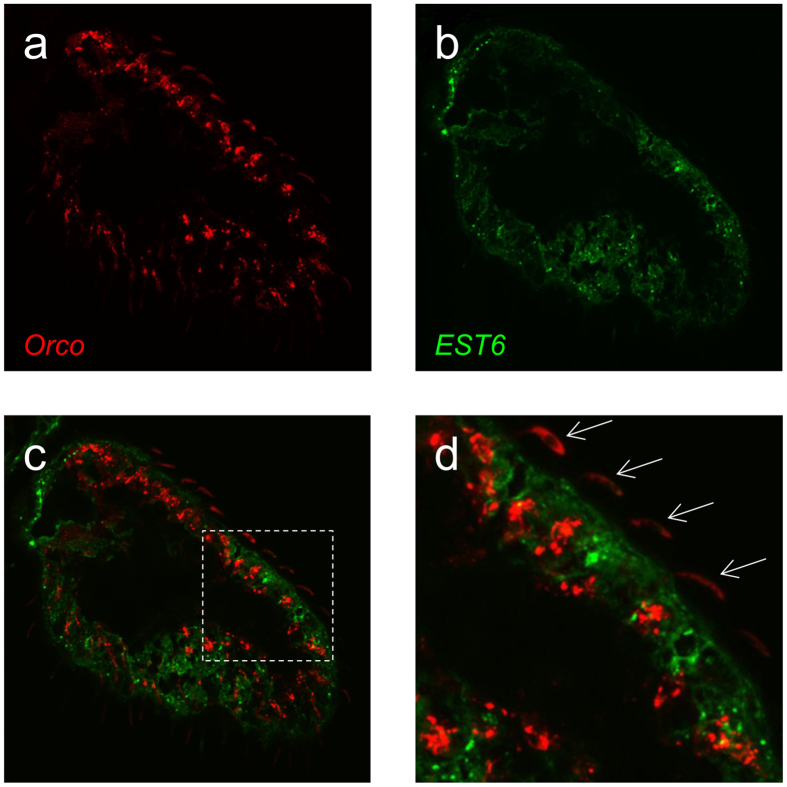
EST6 and *Orco* expression in the third antennal segment, longitudinal sections. (**a**) Membrane-tethered RFP expressed with the *Orco* promoter (*Orco*^*Gal4*^ */UAS-mCD8::RFP* transgenic flies). (**b**) EST6 protein localization in the same section. (**c**) Merge image of (**a**,**b**): Est-6 and *Orco* are not expressed in the same cells. (**d**) Higher magnifications of (**c**): EST6 protein surrounds the Orco^^+^^ dendrites. Arrows indicate the dendrites of *Orco* expressing ORNs. Western blots and immunohistochemistry showing the specificity of the anti-EST6 antibody are shown in Supplementary Fig. S6.

*Est6* is known to be highly expressed in the antenna^8^, in particular in the third antennal segment^10^, but its expression in this tissue at the cellular level was unknown. Labelling of EST6 with anti-EST6 antibody and of Red Fluorescent Protein (RFP) under the control of the *Orco* promoter (*Orco* encodes the universal odorant co-receptor Orco) in transgenic adults showed EST6 immunoreactivity in numerous cells at the base of olfactory sensilla throughout the third antennal segment whereas, as expected^42^, the *Orco* promoter directed expression in numerous olfactory receptor neurons (ORNs) and in cilia entering the sensillar lumen (Fig. 4). As was earlier suggested by Chertemps *et al*.^10^, there was thus no co-localization of the two signals, showing that EST6 is not expressed in ORNs. Similarly, a complementary experiment showed no co-localization of EST6 and the neuron-specific expression of Green Fluorescent Protein (GFP) under the control of the *elav* promoter^43^ (Supplementary Fig. S7). Given that EST6 is a secreted enzyme, this confirms that the enzyme surrounds the Orco^+^ dendrites within the sensillar lymph of various sensilla.

Co-labelling of EST6 and *lush* was then performed to investigate whether the location of EST6 in the sensillar lymph includes the T1 trichoid sensilla involved in cVA detection. LUSH is known to be expressed in all trichoid types^44^. Labelling of EST6 with anti-EST6 antibody and of RFP under the control of the *lush* promoter in transgenic adults found that both signals were closely associated but with no co-localization of the two. RFP was found at the base of trichoid sensilla in accessory cells (Supplementary Fig. S8) that could correspond to trichogen and tormogen cells^44^, whereas EST6 was apparently produced by different support cells for the trichoid sensilla than the LUSH-producing cells^45^, and possibly also by the epidermal cells surrounding the sensilla. To corroborate this result we also performed RNAi knock-down experiments. These results are also consistent with *Est6* is not being co-expressed with *lush* (Supplementary text).

Altogether, these data show that EST6 is produced by non-neuronal cells in the olfactory sensilla, most probably in a large population of accessory cells surrounding ORNs. It localization in the sensillar lymph is compatible with a function of a general ODE in the basiconic sensilla involved in the detection of almost all the substrates tested here^46^. Its function in the T1 trichoid sensilla is not yet clear but its effect on cVA processing in the absence of any direct hydrolytic activity for the compound may reflect a general scavenging role for other ester odorants which might otherwise impede the processing of cVA by its own, as yet unknown, ODE. It is possible that it also plays an equivalent broad scavenging role in some of the other sensilla where it is abundant, although its strong hydrolytic activity for many ester kairomones suggests it has a direct ODE function for several of them.

## Discussion

Notwithstanding the genetic evidence that EST6 contributes to cVA processing *in vivo*^10^, we find that the enzyme has negligible activity (<1.5 M^−1^.s^−1^) for this substrate *in vitro*, with or without LUSH in the assay mix. Our results in fact confirm the only other direct measure of its *in vitro* activity, by Mane *et al*.^9^; their estimation of 55 picomoles of cVA per min per g of purified EST6, or 3.4 M. min^−1^.M^−1^, (in the absence of LUSH) is in the range that was too low to measure accurately in our assays. We concur with Vandermeer *et al*.^17^ that activity in this range is most unlikely to be physiologically relevant. This indicates that the *in vivo* effects of EST6 on cVA processing seen by Chertemps *et al*.^10^ must be indirect.

While we found that EST6 had low activity against cVA, it clearly has physiologically significant (*k*_cat_/*K*_M_ > 10^5^ M^−1^.s^−1^)^25^ activity with a wide range of esters with acyl chains up to six carbons in length and alcohol groups from mid length (3–10 carbon atoms), aliphatic moieties to branched, secondary, unsaturated, cyclic and aromatic groups. These substrates include many fruit and yeast volatiles that are known to be bioactive against *Drosophila*, consistent with the results of electrophysiological and behavioural comparisons of wildtype and EST null flies by Chertemps *et al*.^10^, which show that the enzyme contributes to the processing of many such molecules *in vivo*. As such, our biochemical data support the proposition that EST6 is a general, rather than specific, odorant degrading enzyme (ODE), but with a substrate range tuned to various volatile esters with relatively short chain acyl groups that are commonly emitted by the food sources for the flies.

Significantly, the bioactivity of many of these better substrates for EST6 involves attraction behaviours^47^. For example, fruity smelling acetate esters such as isopentyl and pentyl acetate, which are produced by both plants and yeasts, are highly attractive to *Drosophila*^48^, wherein they activate several fairly broadly tuned odorant receptors, such as Or43b, Or47a and Or85b^46^,^49^. Likewise, the phenolic yeast volatile phenethyl acetate elicits an attraction response from the fly^50^ and activates its Or85d receptor^49^. Notably, some of these attraction behaviors also manifest as effects on reproductive traits; for example, citrus fruits emit many short-mid chain volatile acetates (e.g. propyl, hexyl, heptyl, nonyl, decyl, neryl and geranyl acetates^51^), which attract females to lay eggs^28^.

It has been shown that several food odors, including ester substrates for EST6, can act synergistically with cVA in both aggregation and courtship bioassays^52^,^53^,^54^,^55^. Indeed, some evidence suggests that cVA only acts as an aggregation pheromone in the presence of attractive food odors^55^. It is suggested that the co-processing of pheromonal and kairomonal stimuli would help coordinate feeding and oviposition site selection with reproductive behaviors^53^. However, we cannot see how this synergism would explain the indirect effects of EST6 activity on cVA processing observed by Chertemps *et al*.^10^. One reason is that the experimental design of that previous study meant that food odors would not have been present in the cVA atmospheres they tested. Furthermore, the co-processing of the signals from cVA and the food odors must occur downstream of their receptors, since they have different receptors and the signals from their receptors are transmitted to different glomeruli in the brain, but the effects of EST6 on EAG responses to cVA seen by Chertemps *et al*.^10^ must occur prior to or at the time when the cVA interacts with its receptors. Other indirect effects of EST6 on cVA processing must therefore explain the data of Chertemps *et al*.^10^. For example, as noted above, EST6 may facilitate cVA processing simply by removing other potential substrates (or inhibitors) of the ODE that does degrade cVA. As noted, the latter ODE may be a lipase, and indeed, with an 18-carbon leaving group, cVA is more like a typical lipase substrate than an esterase substrate. Our localization studies would certainly allow for that, given the broad distribution of the enzyme through the sensillar lymph. Further work is needed to elucidate the molecular basis for the effects seen by Chertemps *et al*.^13^.

Our biochemical studies also bear on the question of the molecular basis for the effects on female oviposition and remating behaviors due to the ejaculatory duct EST6 transferred from their mates^15^,^16^. This enzyme is known to be transferred from the female’s reproductive tract to her haemolymph within minutes of mating^14^, but its fate from there and its substrate in the female are unknown. Our results indicate that a wide variety of esters of terpene or aromatic alcohol groups and short-mid chain acids could be candidate substrates. Notably, some such compounds are precursors for various hormones and other key molecules in the fly^56^,^57^. Modern metabolomic technologies may be useful in identifying the *in vivo* substrate for the transferred EST6, particularly given the availability of the *Est6°* flies and wildtype revertants on the same genetic background^10^.

EST6, in Clade E of the carboxylcholinesterase gene family, is not closely related in sequence (26–29% amino acid identity) to any of the three insect esterases for which structures have been solved previously (in Clades B, G and J). While it’s overall structure is similar to the other three, we noted several significant differences in relation to its active site. Of particular note was the appearance of an active site entrance on the opposite face of the protein to that containing the active site entrance in the other three structures. Interestingly, the entrance in EST6 corresponds to the alternative ‘back door’ entrance that has been proposed for AChE^58^. Moreover, the corresponding surface of the catalytically inactive ligand-binding ‘esterase’ neuroligin is the site to which its ligand binds^59^.

Transcriptomic analyses of sensory tissues in various insects have shown as many as half of the catalytically competent carboxyl/cholinesterases in some insects may be expressed at readily detectable levels in their sensory tissues^8^,^60^. The few for which there is any empirical support for ODE functions have spanned four major Clades (A, D, E and G)^18^,^61^, suggesting that esterase ODEs may have evolved independently on several occasions. However, there is a concentration of putative esterase ODEs in the particular lineage within Clade E that contains EST6 (31% amino acid identity)^18^. This lineage contains esterases from at least four insect orders, including one of the best-understood ODE’s at a physiological level, the Apo1PDE from the silkmoth *Antheraea polyphemus*. Apo1PDE is highly specific ODE for a particular sex pheromone substrate^62^, whereas we find EST6 has both broad activity for many kairomones and an indirect effect on cVA processing whose mechanism we currently do not understand. Further work on this lineage could elucidate a range of biochemical, physiological and evolutionary phenomena concerning the function of esterases in insect antennae.

## Methods

### EST6 activity assays

The expression of wildtype EST6 and an inactive EST6 variant in the baculovirus system has been described previously^13^. These two enzymes were assayed here for activity against 85 ester odorants of potential ecological relevance^49^,^63^,^64^ and two other model substrates (listed in Supplementary Table S1). All these esters were purchased in the highest available purity.

Eighty two of the esters were first subjected individually to gas chromatography-mass spectrometry (GC-MS, 7890 series, Agilent Technologies, USA) to determine their respective retention times. A J&W DB-WAX column (30 m × 0.25 mm × 0.25 μm, Agilent Technologies, USA) was used with He (2 ml. min^−1^) as the carrier gas. The oven temperature was initially set at 50 °C for 2 mins and then subsequently increased over a gradient of 10 °C to 275 °C and held for 10 mins. The injector and detector temperature was set at 250 °C with a 10:1 split ratio.

Mixtures of up to 17 compounds with non-overlapping GC-MS retention times were then made in Tris HCl buffer pH 8.0 for a set of preliminary ‘group assays’. Each group included pentyl acetate as a common ester substrate standard. All compounds had been dissolved in ethanol to give a 5% *v/v* final solvent concentration; preliminary assays on some of the more water-soluble esters showed that this ethanol concentration increased *K*_M_ by 5–20 fold (see below) but lower concentrations of ethanol were insufficient to solubilize some compounds and equivalent concentrations of other organic solvents tested were more disruptive to EST6 activity. Several reactions were set up at 25 °C in Tris-HCl buffer pH 8.0 with each ester in the mixture at a final concentration of 200 μM and the enzyme (added last) at 8.2 nM. Individual reactions were then stopped by the addition of 0.5 volumes of ice-cold hexane containing 200 μM heptanone as an internal non-ester standard at intervals from 5 to 65 mins. The concentrations of the various esters remaining were then determined by GC-MS as above. EST6 activity was calculated from the difference in substrate usage between the wildtype and null enzymes, but all values for the latter were essentially negligible.

Subsequently, 43 substrates from the group assay, including all the better substrates, were assayed individually in order to obtain estimates of *k*_cat_/*K*_M_ using equation (1):


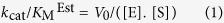


where [E] and [S] are the starting enzyme and substrate concentrations respectively, and *V*_*0*_ is the initial velocity of the reaction^65^.

Aside from the single substrate, these assays were the same as those for the group assays, except that a lower enzyme concentration was used (0.1 to 3.6 nM). The appropriate enzyme concentration was inferred from the enzyme’s activity towards each substrate in the group assay. Three other esters that were not included in the group assays but with closely similar chain lengths and structures to some of the best substrates were also assayed individually in this way. Individual substrate assays with two model esters, 4 NPA and 2 NA, were also carried out using previously described 420 and 390 nm UV/vis protocols for monitoring substrate loss^8^,^21^. *K*_M_ estimates could be obtained from these data for a few substrates and a few were also obtained using the competitive inhibition method with 4 NPA as substrate as described in Younus *et al*.^8^. All the above assays were conducted in triplicate.

### Assays with LUSH

Some assays were also conducted in the presence of the odorant binding protein LUSH. In preparation for this the *lush* coding region was synthesized by Invitrogen and cloned into the expression vector pETMCSI^66^. The LUSH protein was overexpressed in inclusion bodies of *E. coli* BL21 (DE3) star (Invitrogen) cells after overnight growth in Lysogeny Broth (LB) broth containing 100 mg. l^−1^ of ampicillin at 37 °C. The cells were harvested by centrifugation at 5,000 *g* for 5 min at 4 °C, the cells lysed by three passages through a French Press, and the inclusion bodies collected by centrifugation at 10,000 *g* for 20 min at 4 °C. The inclusion bodies were then solubilized and refolded following the method of Kruse *et al*.^67^ using a cysteine-cystine redox reaction in the presence of 1% *v/v* ethanol. The only modifications to this method were that 8 M urea was used to solubilize the inclusion bodies and the soluble protein was dialyzed in 20 mM Tris pH 7.4, 50 mM NaCl. The soluble LUSH was further purified by using a Superdex 200 preparation size exclusion column (GE Healthcare, UK) and assayed for binding activity with the model ligand N-phenyl-1-naphthylamine (NPN) according to the method described by Katti *et al*.^68^. This involved titrating LUSH (1 μM) with increasing amounts of NPN to final concentrations ranging from 0.5 μM to 20 μM. A saturable NPN fluorescence change was recorded by a fluorometer and the dissociation constant was found to be 2.39 μM. Katti *et al*.^68^ showed that LUSH does not display a saturable NPN fluorescence change if it is not fully refolded.

Assays to investigate the activity of EST6 towards cVA in the presence of LUSH were set up the same as those for the group assays except for changes to the substrate (150 μM) and enzyme (3 and 57 nM) concentrations, and the addition of LUSH (300 μM). Duplicate reaction mixtures were set up without LUSH as controls. Equivalent reactions using a better, mid-chain ester substrate, decyl acetate, were also set up as further controls.

### Protein engineering and expression

Six generations of directed evolution were undertaken to improve the soluble expression of *E. coli*-expressed wildtype EST6. The method followed Jackson *et al*.^21^, but in this case the coding region of *Est6* from the iso-1 *y*^*1*^*cn*^*1*^*bw*^*1*^*sp*^*1*^ reference strain (http://flybase.org/reports/FBsn0000272.html), omitting the 63 bp encoding the N-terminal signal peptide^33^, was cloned into the expression vector pETMCSIII^66^ between the *Nde*I and *Eco*RI sites in frame with the ATG start codon of the *Nde*I site. Adequate expression of *Est6* could be achieved by ‘leaky expression’ because of the presence of trace amounts of lactose in the LB media used. The error-prone PCR protocol used to construct the initial mutant library involved a reaction mixture comprising 100–200 ng of pETMCSIII-*Est6*, 1 μM primers pET3 and pET4 (5′CGACTCACTATAGGGAGACCACCAC3′ and 5′CCTTTCGGGCTTTGTTAGCAG3′), 1 × Taq DNA polymerase buffer, 5 mM MgCl_2_, 0.1–0.4 mM MnCl_2_, 0.5 mM dNTPs, 5U Taq DNA polymerase, and milliQ H_2_O to a final volume of 50 μl. Thermocycling involved 30 cycles of 94 °C for 10 s, 45 °C for 10 s and 30 s at 72 °C. The *Nde*I- and *Eco*RI-digested PCR product was gel extracted, ligated back into pETMCSIII, and then used to transform competent BL21 (DE3) star cells. Transformed cells were plated onto LB plates containing 100 mg. l^−1^ ampicillin. After incubation at 30 °C overnight, the colonies were blotted onto 3 M filter papers and esterase activity was assayed by staining the filter paper with a solution consisting of 10 ml of 0.1% *w/v* Fast Red and 0.2 ml of 1% *w/v* 2 NA in 0.1 M Tris pH 7.0. Between 200–300 (approximately 1%) of the colonies generating the most intense red colour were then picked by hand and grown overnight in 500 μl of LB, 100 mg. l^−1^ ampicillin, in 96-well culture plates. 50 μl of each of these cultures was then added to the corresponding well of a 96-well assay plate that contained 250 μl of a reaction mixture consisting of 0.5 mM 2 NA, 0.5 mM Fast Red, and 0.1 M Tris pH 7.0. The reaction was monitored with a spectrophotometer at 490 nm, and the 10–20 colonies generating the highest activities were sequenced and used as parents for the next generation of mutation and selection. The protocols for generations 2 to 6 followed those above. The sixth generation mutant generating the highest activity in the spectrophotometric assay, denoted EST6-1, was used for crystallization.

### EST6-1 Crystallization and Computational Analysis

EST6-1 was expressed in *E. coli* Origami B (DE3) pLysS Cells (Merck) grown in LB media with 100 μg.ml^−1^ ampicillin to an optical density of 0.6. The cells were induced with 700 μM IPTG and harvested after 18 hours at 25 °C. The cells were then lysed by sonication in 50 mM Hepes (pH 7.5), 300 mM NaCl, 10 mM imidazole (buffer A). The soluble lysate was separated by centrifugation at 23,000 *g* and filtered with a 0.45 μM filter before being loaded onto a 5 ml Ni-NTA column. The protein was eluted from the column with buffer A supplemented with 300 mM imidazole. Fractions were pooled after confirmation by SDS-PAGE and further purified by size exclusion chromatography in 20 mM Hepes (pH 7.5) and 50 mM NaCl (buffer B) using a Hiload 26/600 Superdex 200 pg column (GE Healthcare). The concentration of EST6-1 was determined at 280 nm with the Nanodrop 1000 (Thermo Scientific) using an extinction coefficient of 74,635 M^−1^ cm^−1^ estimated by the ProtParam server^69^.

Surface lysine residues of purified EST6-1 (1 mg. ml^−1^) were methylated following the protocol of Walter *et al*.^36^, and the reaction was quenched with 1 M glycine, followed by concentration of methylated EST6-1 to 18.2 mg. ml^−1^ and dialysis into buffer B. Crystals of methylated EST6 were grown by the sitting drop diffusion technique with a reservoir solution containing 0.2 M ammonium acetate, 0.1 M Tris pH 8.5 and 25% *w/v* PEG 3,350. 35% *w/v* PEG 3,350 was used as a cyroprotectant during flash cooling of the crystals in nitrogen at 100 K. Diffraction data were collected at the MX2 beamline at the Australian Synchrotron, Victoria, Australia with a wavelength of 0.9655 Å. Data collection methods and statistics as well as details of the informatics methods used to solve the enzymes structure are given in Supplementary Table S5.

A model EST6-WT was built from the 97% identical structure of EST6-1 using FoldX^40^. Covalent docking was performed with DOCKovalent, a covalent version of DOCK3.7^41^. The program pre-generates a set of conformations for each ligand, covalently attaches the ligand to a receptor, and exhaustively samples ligand orientations around the covalent bond. Ligands are then ranked via a physics-based scoring function. Esters investigated in this work were represented as SMILES strings with the covalent attachment to the catalytic serine Oγ marked with a dummy atom. The esters were docked in the form of a tetrahedral intermediate, after nucleophilic addition of the serine Oγ to the carbonyl carbon and prior to departure of the alcohol, with the carbonyl oxygen bearing a negative charge. The generation of ligand conformations and preparation of the receptor (EST6-WT model) was carried out as described previously^41^. The catalytic histidine was represented in its doubly protonated form. The selected esters were covalently docked onto the Ser188 Oγ with a Oγ-ligand bond length of 1.6 ± 0.1 Å sampled at 0.05 Å increments and with the Cβ-Oγ-ligand and Oγ-ligand-ligand bond angles set to 109.5 ± 5° and sampled at 1° increments. The lowest energy pose for each ligand was selected for analysis. Protein structure images were produced with PyMol V 1.3 and a topology diagram was generated using TOPDRAW^70^.

### Immunohistochemistry

#### Flies

*Orco*^*Gal4*^ flies were generously provided by G. Galizia (University of Konstanz, Germany), *lush*^*Gal4*^ flies (originally from R Benton, Université de Lausanne, Switzerland) from J-F. Ferveur (CSGA, Dijon, France) and *elav*^*LexA*^, *LexAOP-mCD8::GFP, UAS-mCD8::RFP* and *Est6* null mutant flies from the Bloomington Stock Center (stocks 52676, 32203, 27392 and 4211 respectively). All flies were raised at 25 °C on standard yeast/cornmeal/agar medium in a 12-hr light/12-hr dark cycle, 50–60% relative humidity.

#### Generation of anti-EST6 antiserum

Preparation of denatured EST6 antigen and production of polyclonal antibody followed the methods of Han *et al*.^71^. Briefly, wildtype EST6 was overexpressed in inclusion bodies in *E. coli* using the expression vector pETMCS III as above. Cells were harvested and lysed and inclusion bodies collected as above. The latter were then dissolved in 6 M guanidine HCl in a buffer containing 20 mM phosphate pH 7.4 and the solubilized denatured proteins loaded onto a 5 ml Ni-NTA column. The EST6 was eluted from the column with a gradient of buffer containing 6 M guanidine HCl, 20 mM phosphate, 0.5 M imidazole, pH 7.4. Fractions containing EST6 were identified from the presence of a 59.7 kDa band on denaturing PAGE and then pooled and loaded onto a Superdex 200 preparative scale exclusion column (GE Healthcare) equilibrated with 6 M of guanidine HCl, 20 mM phosphate buffer, pH 7.4. EST6 fractions from this column were concentrated to 1 mg. ml^−1^ using an Amicon Ultra-15 centrifugal device (Millipore, US) and the guanidine HCl removed by dialysis in 20 mM phosphate buffer, pH 7.4. The purified denatured EST6 was used as antigen for polyclonal antibody production by IMVS Veterinary Services, South Australia. Four doses of 0.5 mg antigen were administrated to a rabbit at 3 weekly intervals. The polyclonal antibodies were purified from antiserum using an IgG affinity column and the protein concentration was estimated at 3 mg. ml^−1^.

The specificity of the antiserum was then tested by western blotting against extracts from heads of wildtype (*Canton S*) and *Est6°* null mutant flies. Mass homogenates of heads from each strain in 20 mM Tris-HCl buffer (pH 7.4) were briefly sonicated, centrifuged at 8,000 g for 10 min and the supernatants isolated. Twenty μg of protein from each homogenate were then separated by SDS-PAGE and blotted onto a polyvinylidene fluoride (PVDF) membrane. After blocking in Tris Buffered Saline-Tween 10% (TBST-10%) blocking reagent (Invitrogen), membranes were incubated overnight at 4 °C with the anti EST6 antibody (1:3,000), then incubated with rabbit-peroxidase-labelled antibody (1:10,000). Blots were then washed and incubated with chemiluminescent substrate (ECL Plus Western Detection Kit, GE Healthcare).

### Localization of EST6 within antennae

To localize EST6 in the antenna, we performed immunohistochemistry with the anti-EST6 antibody above on transgenic flies expressing RFP under the control of either the *Orco* or *lush* promoter or GFP under the control of the *elav* promoter. *Est6* null mutant flies were used as a control for the specific labelling of the antibody. Specifically, heads with antennae still attached from 5-day-old *Orco*^*Gal4*^*/UAS-mCD8::RFP, elav*^*LexA*^*/LexAOP-mCD8::GFP, lush*^*Gal4*^*/UAS-mCD8::RFP* or *Est6* null mutant males were fixed for 3 h in 4% paraformaldehyde with 0.2% Triton X-100, then washed for 1 h with PBS containing 0.2% Triton X-100 (PBST). The heads were then embedded in Tissue-Tek^TM^ (CellPath) and cryosections (15 μm) were set in cell culture insert (Greiner Bio-one). After blocking with 3% normal goat serum and 1% BSA in PBST (1 h at room temperature), the anti-EST6 antibody was diluted from 1:3,000 to 1:750 (*v:v*) in the blocking solution (3% normal goat serum in PBST) and incubated overnight at room temperature. After a brief rinse in PBST, an anti-mouse conjugated Alexa-488 or Alexa-596 (Invitrogen) was applied at a concentration of 1:800 (*v:v*) in the blocking solution for 4 h at room temperature. Tissues were mounted in Slowfade reagent with DAPI (Invitrogen). Images were captured on a Leica SP5 confocal microscope and analysed using ImageJ 1.47 v (http://imagej.nih.gov/ij).

## Additional Information

**How to cite this article**: Younus, F. *et al*. Molecular basis for the behavioral effects of the odorant degrading enzyme Esterase 6 in *Drosophila.* *Sci. Rep.* **7**, 46188; doi: 10.1038/srep46188 (2017).

**Publisher's note:** Springer Nature remains neutral with regard to jurisdictional claims in published maps and institutional affiliations.

## Supplementary Material

Supplementary Material

## Figures and Tables

**Table 1:** Active site volume calculated using the CASTp server.

Protein	Active Site Volume (Å^3^)	Distance from surface to active site Serine (Å)
EST6 WT FoldX Model	408	15.1
EST6-1 Crystal Structure	935	15.1
*Lc*αE7 (4FNG)	2727	20.2
*Dm*AChE (1QO9)	782	17.2
*Ms*JHE (2FJ0)	1308	18.1
Lipase (1AQL)	3074	17.4
