# Supplementary material for: Molecular basis for the behavioral effects of the odorant degrading enzyme Esterase 6 in Drosophila
Source: Sci Rep. 2017 Apr 10;7:46188. doi: 10.1038/srep46188 (PMC5385555; doi:10.1038/srep46188)
Supplement: Supplementary Material [file srep46188-s1.pdf]

Supplementary material:

Molecular basis for the behavioral effects of the odorant degrading enzyme Esterase 6 in *Drosophila*

Faisal Younus [1,2], Nicholas J. Fraser [2], Chris W. Coppin [1], Jian-Wei Liu [1], Galen J. Correy [2], Thomas Chertemps [3], Gunjan Pandey [1], Martine Maïbèche [3], Colin J. Jackson [2], John G. Oakeshott [1]

[1] CSIRO Land and Water, Black Mountain, Canberra, ACT, 2601, Australia

[2] Research School of Chemistry, Australian National University, Canberra, ACT, 2601, Australia

[3] Université Pierre et Marie Curie, Institut d'Ecologie et des Sciences de l'Environnement de Paris, 75252, Paris, France

a

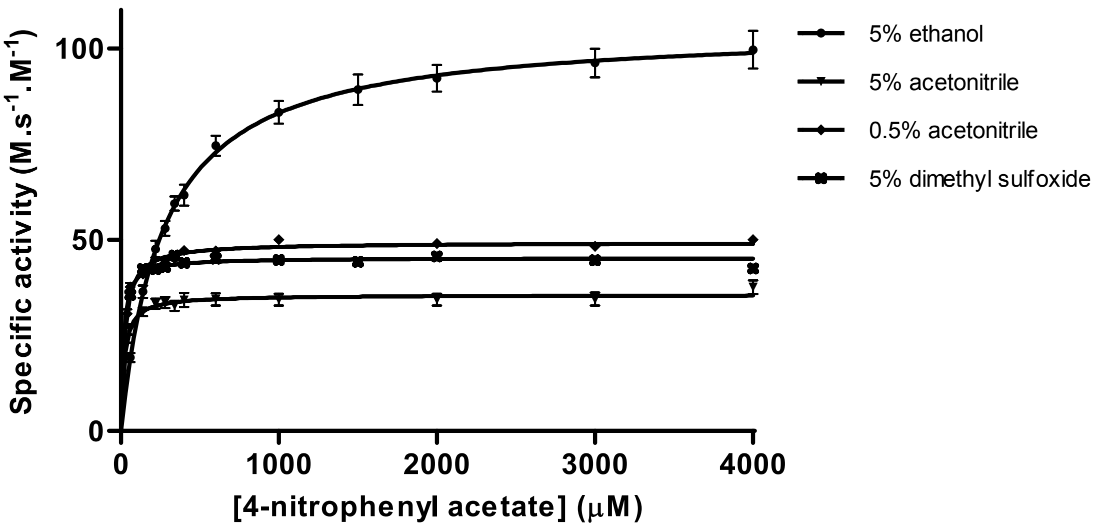

b

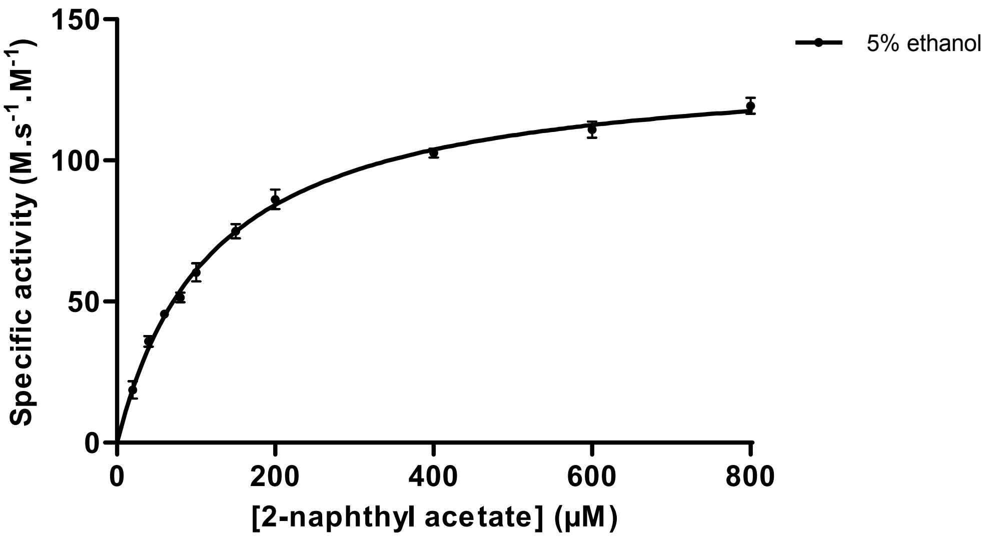

c

Substrate	Solvent conditions	k_{cat} M.s^{-1}	K_M μM	k_{cat}/K_M M^{-1} s^{-1} × 10^3
4-Nitrophenyl acetate	5% ethanol	105 ± 2	269 ± 18	392
	5% acetonitrile	36 ± 1	19 ± 3	1828
	0.5% acetonitrile	49 ± 0	23 ± 1	2111
	5% dimethyl sulfoxide	45 ± 0	14 ± 1	3253
2-Naphthyl acetate	5% ethanol	135 ± 2	121 ± 6	1118

Supplementary Figure S1. EST6 kinetic data towards the model substrates 4-nitrophenyl acetate and 2-naphthyl acetate. **(a)** EST6 Michaelis-Menten plot for 4-nitrophenyl acetate under various solvent conditions. **(b)** EST6 Michaelis-Menten plot for 2-naphthyl acetate. **(c)** EST6 kinetic parameters.

Variant	2	15	50	55	72	75	76	94	98	106	117	119	145	151	180	182	208	219	225	229	237	242	246	247	263	267	271	273	274	285	290	292	302	305	313	329	331	335	336	351	355	357	378	381	382	383	398	400	416	450	456	459	461	462	464	483	484	485	492	496	498	503	508	511	515	517
Wt	D	K	K	I	A	A	N	N	N	I	W	N	V	D	Q	V	R	D	K	G	N	S	S	T	T	K	F	Y	V	P	D	I	K	K	V	K	R	I	V	L	R	K	Q	D	I	E	K	S	A	F	F	D	E	M	P	S	D	N	E	K	N	K	R	I	Q	R
#1-2	.	.	.	.	.	.	.	.	.	.	.	.	.	.	.	.	.	.	.	.	.	.	.	.	.	R	.	.	.	.	.	.	.	.	.	.	.	.	.	.	.	.	.	.	.	.	.	.	.	.	.	.	.	.	.	.	.	.	.	.	S	.	.	.	.	.
#1-3	A	.	.	.	.	.	.	.	.	.	.	.	.	.	.	.	.	.	.	.	.	.	.	.	.	.	.	.	.	.	.	.	.	.	.	.	.	.	.	.	.	.	.	.	.	.	.	.	.	.	.	.	.	.	.	.	.	.	.	.	S	.	.	.	.	.
#1-4	.	.	.	.	.	.	.	.	.	.	.	.	.	.	.	.	.	.	.	.	.	.	.	.	.	.	.	.	.	.	.	.	.	.	.	.	.	.	.	.	.	.	R	.	.	.	.	.	.	.	.	.	.	.	.	.	.	.	.	.	S	.	.	.	.	.
#1-5	.	.	.	.	.	.	.	.	.	.	.	.	.	.	.	.	.	.	.	.	.	.	.	.	.	.	.	.	.	.	.	.	.	.	.	.	.	.	.	.	.	.	.	.	.	.	.	.	.	.	.	.	.	.	.	.	.	.	.	.	S	.	.	.	.	.
#1-6	.	.	.	.	.	.	.	.	.	.	.	.	.	.	.	.	.	.	.	.	.	.	.	.	.	.	.	.	.	.	.	.	.	.	.	.	.	.	.	.	.	.	.	.	.	.	.	.	.	.	.	.	.	.	.	.	.	.	.	.	S	.	.	.	.	.
#2-1	.	.	.	.	.	.	.	.	.	.	.	.	.	.	.	.	.	.	.	.	.	.	.	.	.	.	.	.	.	.	.	.	.	.	.	.	.	.	.	.	.	.	.	.	.	.	.	G	.	.	.	.	.	.	.	.	.	.	.	.	S	.	.	.	.	.
#2-2	.	.	.	.	.	.	.	.	.	.	.	.	.	.	.	.	.	.	.	.	.	.	.	.	.	.	.	.	.	.	.	.	.	.	.	.	.	.	.	.	.	.	.	.	.	.	.	.	.	.	.	.	.	.	.	.	.	.	.	.	S	.	.	T	.	.
#2-3	.	.	.	.	.	.	.	.	.	.	.	.	.	.	.	.	.	.	.	.	.	.	.	.	.	.	.	.	.	.	.	.	.	.	.	.	.	.	.	.	.	.	.	.	.	.	.	.	.	.	.	.	.	.	.	.	.	D	.	.	S	.	.	.	.	.
#2-4	.	.	.	.	.	.	.	.	.	.	.	.	.	.	.	.	.	.	.	.	.	.	.	.	.	.	.	.	.	.	.	.	.	.	.	.	.	.	.	.	.	.	.	.	.	.	.	.	.	.	.	.	.	.	.	.	.	.	.	.	S	.	.	.	.	.
#2-5	.	.	.	.	.	.	.	.	.	.	.	.	.	.	.	.	.	.	.	.	.	.	.	.	.	.	.	.	.	.	.	.	.	.	.	.	.	.	.	.	.	.	.	.	.	.	.	.	.	S	S	.	.	.	.	.	.	.	.	.	S	.	.	.	.	.
#2-6	.	.	.	.	.	.	.	.	.	.	.	.	.	N	.	.	.	.	.	.	.	.	.	.	.	.	.	.	.	.	.	.	.	.	.	.	.	.	.	.	.	.	.	.	.	.	.	.	.	.	.	.	.	.	.	.	.	.	.	.	S	.	.	.	.	.
#2-7	.	M	.	.	.	.	.	.	.	.	.	.	.	.	.	.	.	.	.	.	.	.	.	.	.	.	.	.	.	.	.	.	.	.	.	.	.	.	.	.	.	.	.	.	.	.	.	.	.	.	.	.	.	.	.	.	.	.	.	.	S	.	.	T	.	.
#2-8	.	.	.	.	.	.	.	.	.	.	.	.	.	.	.	.	.	.	.	.	.	.	.	.	.	.	.	.	.	.	G	.	.	.	.	.	.	.	.	.	.	.	.	.	.	.	.	.	.	S	.	.	.	.	.	.	.	.	.	.	S	.	.	.	.	.
#2-9	.	.	.	.	.	.	.	.	.	.	.	.	.	.	.	.	.	.	.	.	.	.	.	.	.	.	.	.	.	.	.	.	.	.	.	.	.	.	.	.	.	.	.	.	.	.	.	.	.	.	.	.	.	.	.	.	.	.	.	.	S	.	.	T	.	.
#2-10	.	.	.	.	.	.	.	.	.	.	.	D	.	.	.	.	.	.	.	.	.	.	.	.	A	.	.	.	.	.	.	.	.	.	.	.	.	.	.	.	.	.	.	.	.	.	.	.	V	.	.	.	.	.	.	.	.	.	.	.	S	.	.	.	.	.
#3-1	V	.	.	.	.	.	.	.	.	.	.	.	L	.	.	.	.	.	.	.	.	.	.	.	.	.	.	.	.	.	.	.	.	.	.	.	.	.	.	.	.	.	.	G	.	.	.	.	.	.	S	.	.	.	.	.	.	.	.	.	S	.	.	T	.	.
#3-2	A	.	.	.	.	.	.	.	.	.	.	.	.	.	.	.	.	.	.	.	.	.	.	.	.	.	.	.	.	.	.	.	.	.	.	.	.	.	.	.	.	.	.	G	.	.	.	.	V	S	S	.	.	.	.	.	.	.	.	.	S	.	.	.	.	.
#3-3	.	M	.	.	.	.	.	.	.	.	.	.	.	.	.	.	.	.	.	.	.	L	.	.	.	.	.	.	.	.	.	.	R	.	.	.	.	.	.	.	.	.	.	G	.	.	.	.	.	S	S	.	G	.	.	.	.	.	.	.	.	.	.	T	.	.
#3-4	A	.	.	.	.	.	.	.	.	.	.	.	.	N	.	.	.	.	.	.	.	.	.	.	.	.	.	.	.	.	.	.	.	.	.	.	.	.	.	.	.	.	D	.	.	.	.	.	.	.	.	.	.	.	.	.	.	.	.	.	.	.	.	.	.	.
#3-5	.	.	.	.	.	.	.	.	.	.	.	D	.	.	.	.	K	.	.	.	.	.	.	.	.	.	.	.	.	.	.	.	.	.	.	.	.	.	.	.	.	.	.	.	.	.	.	.	V	.	S	.	.	.	.	.	.	.	.	.	S	.	.	T	.	.
#3-6	V	.	.	.	.	.	.	.	.	.	.	.	.	.	.	.	.	.	.	.	.	.	.	A	.	.	.	.	.	.	.	.	.	.	.	.	.	.	.	.	.	.	.	.	.	.	.	.	.	.	.	.	.	.	.	.	.	.	.	.	.	.	.	.	.	.
#3-7	A	.	.	.	.	.	.	.	.	.	.	.	.	.	.	.	.	.	.	.	.	L	.	.	.	.	.	.	.	.	.	.	.	.	.	.	.	.	.	.	.	.	.	.	.	.	.	.	V	.	.	.	.	.	.	.	.	.	.	.	.	.	.	.	.	.
#3-8	A	.	.	.	.	.	.	.	.	.	.	.	.	.	.	.	.	.	.	.	.	.	.	.	.	.	.	.	.	.	.	.	.	.	.	.	.	.	.	.	.	.	.	.	.	.	.	.	.	S	.	.	.	.	.	.	.	D	.	.	.	.	.	T	.	K
#3-9	A	.	.	.	.	.	.	.	.	.	.	.	.	.	.	.	K	.	.	.	.	.	.	.	.	.	.	.	.	.	.	.	.	.	.	.	.	.	.	.	Q	.	.	.	.	.	.	.	.	S	.	.	.	.	.	.	.	.	.	.	.	.	.	.	R	.
#3-10	.	.	.	.	.	.	.	.	.	.	.	.	.	.	.	.	.	.	.	.	.	.	.	.	.	.	.	.	.	.	.	.	.	.	A	R	Q	.	.	.	.	.	.	.	.	.	.	G	.	S	.	.	.	.	.	.	.	D	.	.	.	.	.	.	.	.
#4-2	.	.	.	.	.	.	.	.	.	.	.	.	.	.	.	.	K	.	.	.	.	.	.	A	.	.	.	.	.	.	G	F	.	.	.	.	.	V	.	.	.	.	.	.	.	G	.	G	.	S	.	.	.	.	.	.	.	.	.	.	.	.	.	T	.	K
#4-3	.	.	.	.	.	.	.	.	.	.	.	.	.	.	.	.	.	.	.	.	.	.	.	A	.	.	.	.	.	Q	.	.	.	.	.	.	.	.	I	.	.	.	.	.	.	.	.	G	V	S	S	G	.	.	.	.	.	.	.	.	.	.	.	.	.	.
#4-4	.	.	.	.	.	.	.	.	.	.	.	.	L	.	.	.	.	.	.	.	.	.	.	.	.	.	.	.	.	.	.	.	.	.	.	.	.	.	.	.	.	.	.	.	.	.	.	G	.	S	S	.	.	.	.	.	.	.	.	.	.	.	.	.	R	.
#4-5	A	V	.	.	.	.	.	.	.	V	.	.	.	.	.	.	.	.	.	.	.	.	.	.	.	.	.	.	.	.	.	.	.	.	.	.	.	.	.	.	.	.	.	.	.	.	.	.	.	.	S	.	.	.	.	.	.	D	.	.	.	.	.	T	.	.
#4-6	A	.	.	.	.	.	.	.	.	.	.	.	.	.	.	.	.	.	.	.	.	.	.	A	.	.	.	.	A	.	.	.	.	.	.	.	.	.	.	.	.	.	.	G	.	.	.	G	.	S	.	.	.	.	.	.	.	.	.	.	.	.	.	T	.	.
#4-7	.	.	.	.	.	.	.	.	.	.	.	.	L	.	.	I	K	.	.	.	.	.	.	A	.	.	.	.	.	.	.	.	R	.	.	.	.	.	.	.	.	.	.	.	.	.	.	.	V	S	.	.	.	.	.	.	.	.	.	.	.	.	.	T	.	.
#4-8	.	.	E	.	.	.	.	.	.	.	.	.	.	.	.	.	.	.	.	.	.	L	.	A	.	.	.	.	.	.	.	.	.	.	.	.	.	.	.	.	.	.	.	.	.	.	.	.	.	.	.	.	.	.	.	.	.	.	G	.	.	.	.	T	.	.
#4-9	.	.	.	.	.	.	.	T	.	.	.	.	.	.	R	.	K	.	.	.	.	.	.	.	.	.	.	.	.	.	.	.	.	.	.	.	.	.	I	.	.	.	D	.	L	.	.	.	.	.	S	.	.	.	.	G	.	D	.	.	.	.	.	T	.	.
#4-10	.	.	.	.	.	T	.	.	.	.	.	.	.	.	.	.	K	.	.	.	.	.	.	.	.	.	.	.	.	.	G	F	.	.	.	.	.	.	.	.	.	.	.	.	.	G	R	G	V	S	S	.	.	.	L	.	.	.	G	.	.	.	G	T	.	G
#4-11	A	M	.	.	.	T	.	.	.	.	.	.	L	.	.	.	.	.	.	.	.	.	P	A	.	.	.	.	.	.	.	.	.	.	.	.	.	.	.	.	.	.	.	G	.	.	.	G	V	S	.	.	.	V	.	.	.	.	.	.	.	Q	.	T	.	.
#5-1	.	.	.	.	.	.	.	.	.	.	.	.	L	.	.	.	.	.	.	.	.	.	.	A	.	.	.	.	.	.	G	.	.	.	.	.	.	.	.	.	.	.	.	G	.	.	.	.	V	S	S	.	.	.	.	.	.	D	.	.	.	.	.	T	.	.
#5-2	.	.	.	.	.	.	.	.	.	.	.	D	L	.	.	.	.	.	.	.	.	.	.	A	.	.	S	.	.	.	G	.	.	.	.	.	.	.	I	.	.	.	.	.	.	G	.	G	V	S	S	.	.	.	.	.	.	D	.	.	.	Q	.	T	.	E
#5-3	.	.	.	.	.	T	.	.	.	.	.	.	L	.	.	.	K	.	.	.	.	L	.	A	.	.	.	.	.	.	G	.	.	.	.	.	.	.	.	.	.	.	.	.	.	.	.	G	V	S	.	.	.	.	.	.	.	D	.	.	.	.	.	T	.	E
#5-4	.	.	.	.	.	T	D	.	D	.	.	.	L	.	.	.	K	.	.	.	.	.	.	A	.	.	.	.	.	.	.	.	.	.	.	.	.	V	.	.	.	.	.	G	.	.	.	G	V	S	.	.	.	.	.	.	.	D	G	.	.	.	.	T	.	K
#5-5	.	.	.	.	.	.	.	.	.	.	.	.	L	.	.	.	.	.	.	.	.	.	.	A	.	.	.	.	.	.	G	.	.	.	.	.	.	.	.	.	.	.	.	G	.	.	.	G	V	S	S	.	.	.	.	.	.	D	.	.	.	.	.	T	R	.
#5-6	.	.	.	V	.	.	.	.	.	.	.	D	L	.	.	.	K	.	.	E	.	.	.	A	.	.	.	.	.	.	.	.	.	.	.	.	.	.	.	.	.	.	.	.	.	.	.	G	V	S	.	.	.	.	L	.	.	.	.	.	.	.	.	T	.	.
#5-7	.	V	.	.	.	.	.	.	.	.	.	D	L	.	.	.	K	.	.	.	.	.	.	A	.	.	.	.	.	.	.	.	.	.	.	.	.	.	.	.	.	.	.	.	.	.	.	G	V	S	S	.	.	.	.	.	.	.	.	.	.	.	.	.	.	.
#5-8	.	V	.	.	.	.	.	.	.	.	.	.	L	.	.	.	K	.	E	E	.	.	.	A	.	.	.	.	.	.	.	.	.	.	.	.	.	V	.	.	.	.	.	G	.	.	.	G	V	S	.	.	.	.	.	.	.	.	.	.	.	.	.	T	.	G
#5-9	.	.	.	.	.	.	.	.	.	.	.	.	L	.	.	.	.	.	.	E	.	.	.	A	.	.	.	.	.	.	G	.	.	.	.	.	.	.	.	.	.	.	.	.	.	.	.	G	V	S	S	.	.	V	.	.	.	.	G	.	.	.	.	T	.	.
#5-10	.	M	.	.	T	.	.	.	.	.	.	.	L	.	.	.	K	.	.	.	.	.	.	A	.	.	.	.	.	.	.	.	.	.	A	.	.	.	.	.	.	.	.	G	.	.	.	G	V	S	.	.	.	.	.	.	.	D	.	.	.	.	.	T	.	.
#6-1	.	V	.	.	.	.	.	.	.	.	.	.	L	.	.	.	K	.	.	E	S	.	.	A	.	.	.	.	.	.	G	F	.	.	.	.	.	V	.	.	.	.	.	.	.	G	.	G	V	S	S	.	.	.	.	.	.	D	.	.	.	.	.	T	.	.

Supplementary Figure S2. Sample of EST6 replacement changes observed over six rounds of directed evolution. Variable amino acid positions and the status of EST6 Wt are indicated along the top with amino acids numbered from the first residue of the mature EST6 protein as it would be processed in its native form within the fly. Sequence variation in the six rounds of random mutagenesis and directed evolution are indicated below where a dot "." indicates no change. Variant #6.1, which was used for the crystallography, is simply labelled EST6-1 in the main text.

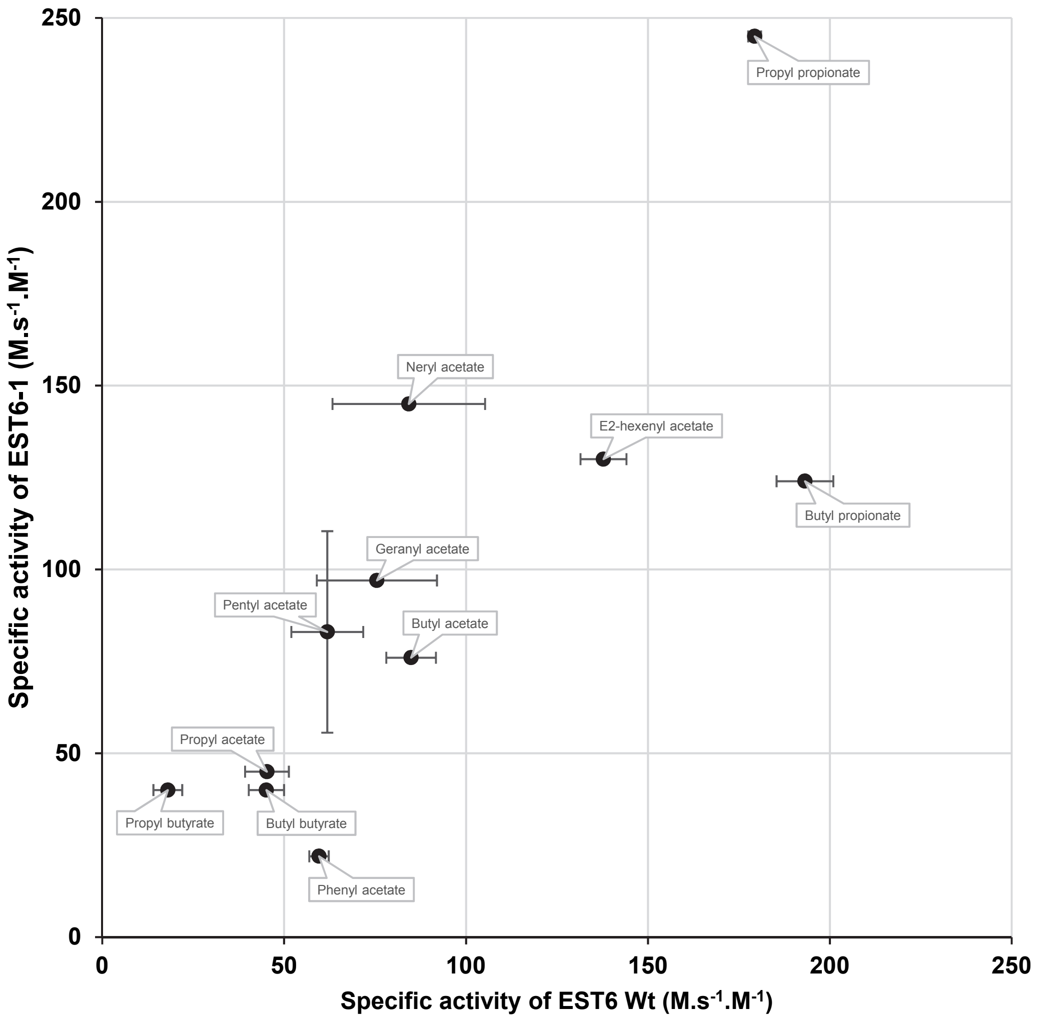

Supplementary Figure S3. Comparison of the catalytic activities of wildtype and evolved EST6 against 11 substrates. Specific activities at 200 µM substrate of wildtype EST6 compared to the evolved EST6-1.

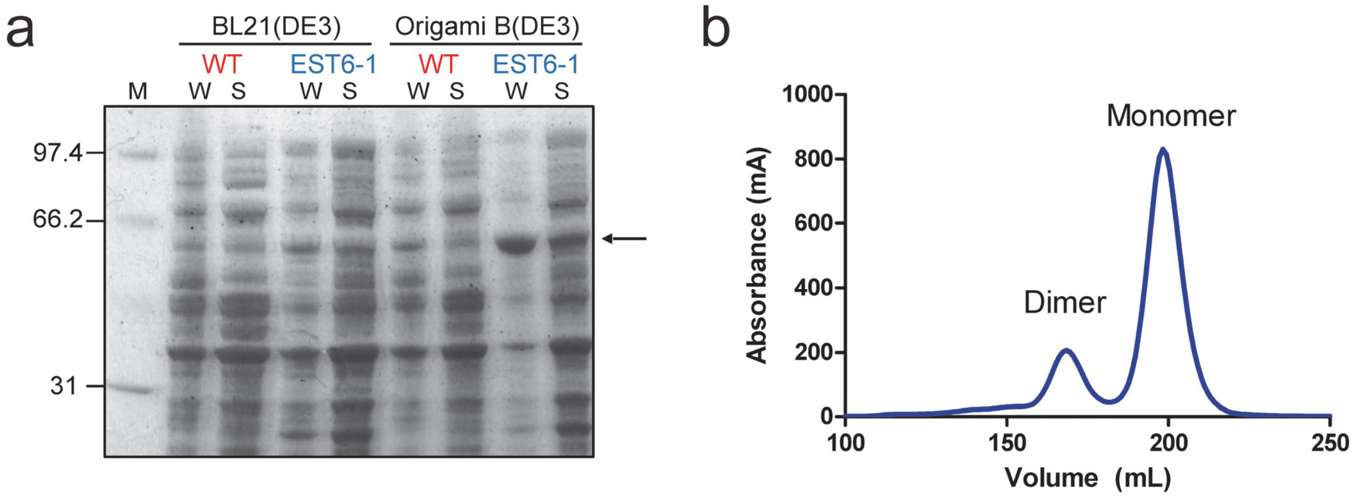

Supplementary Figure S4. Expression and purification of EST6-1. **(a)** Expression gel of whole cell (W) and soluble (S) EST6WT and EST6-1 in the BL21 expression strain and the Origami B expression strain with soluble EST6-1 marked with an arrow. M, molecular markers with sizes indicated in kDa. **(b)** Size exclusion chromatogram of EST6-1 displaying the predominant monomer species used for crystallization.

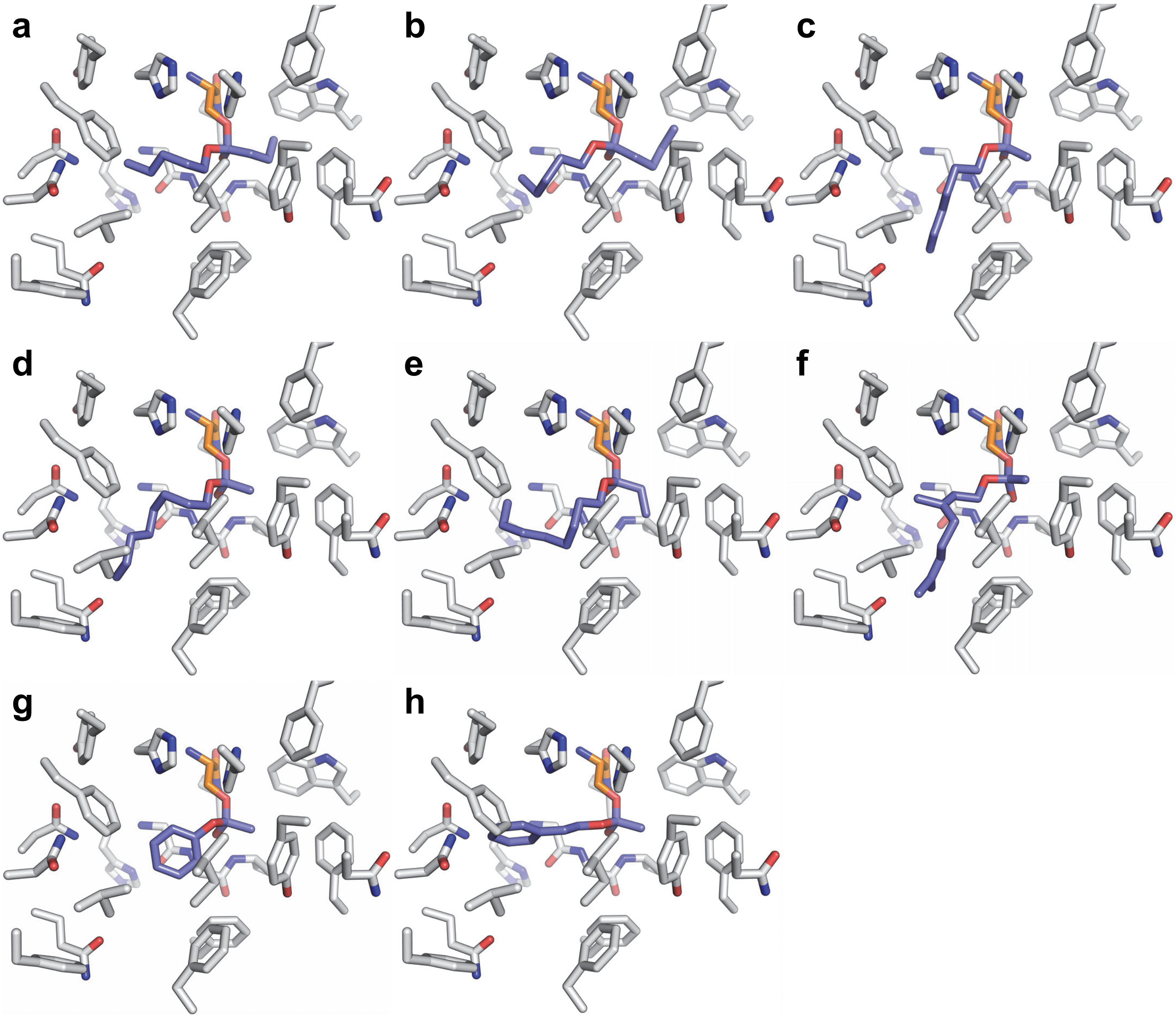

Supplementary Figure S5. Docking of representative substrates in EST6. A series of representative substrates/acyl intermediates were covalently docked into EST6: **(a)** pentyl butyrate, **(b)** pentyl pentanoate, **(c)** hexyl acetate, **(d)** octyl acetate, **(e)** octyl propionate, **(f)** geranyl acetate, **(g)** phenethyl acetate, **(h)** phenyl acetate.

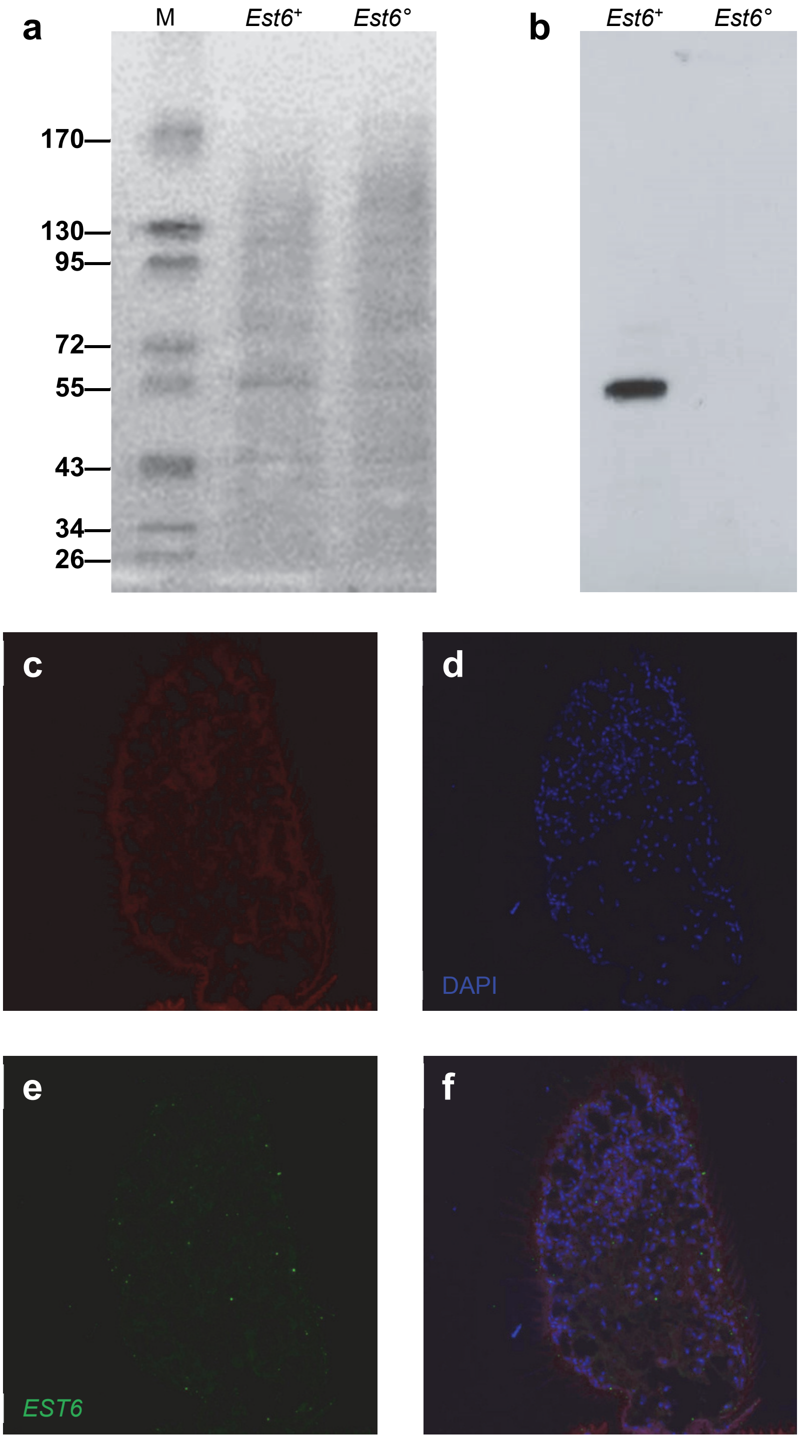

Supplementary Figure S6. Western blot and immunohistochemistry with anti-EST6 antibody showing its specificity for EST6. **(a)** Protein staining of head extracts from wildtype (*Est6*+) and *Est6* null mutant (*Est6*°) flies separated by SDS –PAGE. M, molecular markers with sizes indicated in kDa. **(b)** Same samples showing the location of the bound anti-EST6 antibody. (**a**) Section from third antennal segment of *Est6* null mutant showing autofluorescence of the cuticle. **(b)** DAPI staining of the nuclei. **(c)** EST6 localization on the same section: no labelling with anti-EST6 antibody was detected. **(d)** Merge image of (a), (b) and (c).

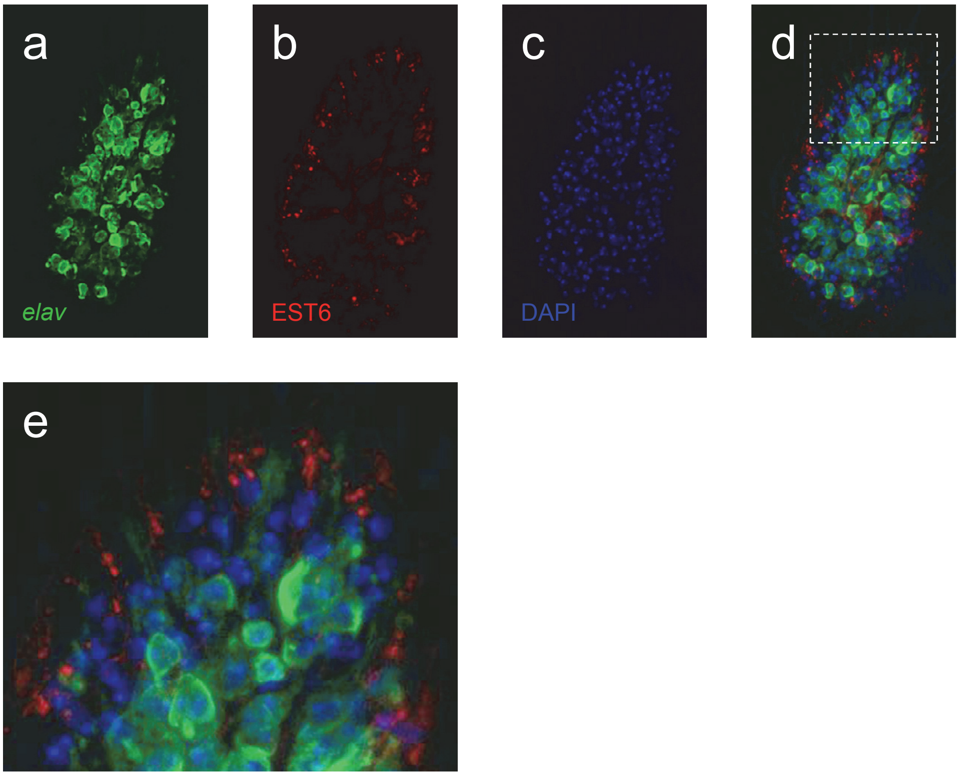

Supplementary Figure S7. EST6 and *elav* expression in the third antennal segment. **(a)** Membrane-tethered GFP expressed with the *elav* promoter (*elav*^{LexA}*/LexAOP-mCD8::GFP* transgenic flies). **(b)** EST6 protein localization in the same section. **(c)** DAPI staining of the nuclei. **(d)** Merge image of (a), (b) and (c): Est-6 and *elav* are not expressed in the same cells. **(e)** Higher magnifications of (c): EST6 protein surrounds the *elav*$^{+}$ dendrites.

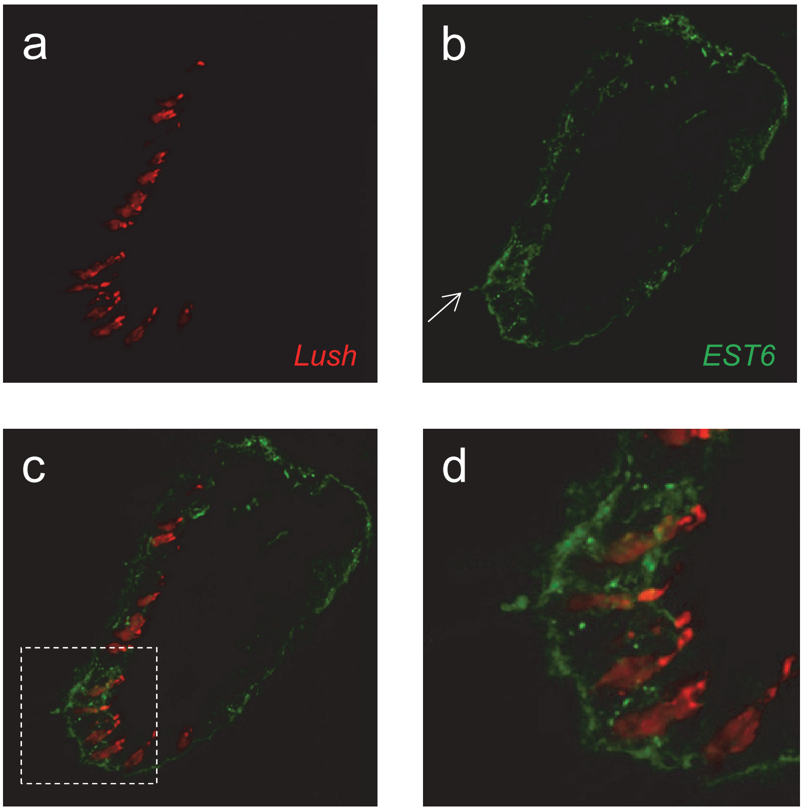

Supplementary Figure S8. EST6 and *Lush* expression in the third antennal segment, transversal sections. **(a)** Membrane-tethered RFP expressed with the *lush* promoter (*lush^{Gal4}/UAS-mCD8::RFP* transgenic flies). **(b)** EST6 protein localization in the same section. Arrow indicate sensillum. **(c)** Merge image of (a) and (b): EST6 and *lush* are not expressed in the same cells. **(d)** Higher magnifications of (c): EST6 protein is secreted in the sensillum lymph.

Supplementary Table S1. EST6 kinetic parameters.

Substrate	$k_{cat}/K_M^{Est‡}$ $M^{-1}.s^{-1}$ × 10^3	K_i µM
**Straight chain esters**		
Methyl decanoate	22 ± 5	
Methyl myristate	15 ± 8	
Propyl acetate	226 ± 30	
Propyl propionate	897 ± 9	
Propyl butyrate	90 ± 20	
Propyl hexanoate	276 ± 25	
Propyl decanoate	28 ± 10	
Butyl formate	472 ± 38	
Butyl acetate	425 ± 34	
Butyl propionate	966 ± 39	
Butyl butyrate	226 ± 24	
Butyl pentanoate	210 ± 18	
Butyl decanoate	109 ± 13	
Pentyl formate	416 ± 38	
Pentyl acetate	309 ± 49	
Pentyl butyrate	185 ± 15	
Pentyl pentanoate	150 ± 20	
Pentyl hexanoate	271 ± 31	
Hexyl formate	309 ± 27	
Hexyl acetate	540 ± 21	
Hexyl propionate	1055 ± 15	
Hexyl butyrate	332 ± 23	
Hexyl hexanoate	72 ± 36	
Heptyl acetate	579 ± 42	
Heptyl propionate	577 ± 124	
Heptyl butyrate	200 ± 15	
Octyl acetate	416 ± 23	
Octyl propionate	1343 ± 12	
Octyl butyrate	365 ± 28	
Nonyl acetate	698 ± 38	
Decyl acetate	740 ± 19	
**Cyclic alcohol moieties**		
Cyclohexyl acetate	102 ± 8	
Benzyl formate	156 ± 42	
Benzyl acetate	328 ± 22	430 ± 40
Phenyl acetate	298 ± 13	190 ± 0
Phenethyl acetate	142 ± 9	880 ± 40
4-Nitrophenyl acetate*	224 •	
2-Naphthyl acetate*	399 •	
**Branched and unsaturated alcohol moieties**		
Isobutyl acetate	369 ± 48	
Isopentyl acetate	315 ± 38	
2-Methylbutyl acetate	213 ± 18	
cis-3-Hexenyl acetate	295 ± 32	
E2-hexenyl acetate	689 ± 32	
Geranyl formate	375 ± 42	
Geranyl acetate	378 ± 83	
Neryl acetate	421 ± 105	
**Modified acid moieties**		
Butyl 2-methylbutyrate†	26 ± 8	
Hexyl 2-methylbutyrate	220 ± 29	

† k_{cat}/K_M^{Est} was taken from group assay results.
‡ k_{cat}/K_M^{Est} was below 1.5 x 10^4 for the following substrates: methyl propionate, methyl butyrate, methyl pentanoate, methyl hexanoate, methyl heptanoate, methyl octanoate, methyl laurate, methyl palmitate, ethyl propionate, ethyl butyrate, ethyl pentanoate, ethyl hexanoate, ethyl heptanoate, ethyl octanoate, ethyl decanoate, linalyl acetate, cis-vaccenyl acetate, ethyl lactate, methyl 3-hydroxyhexanoate, ethyl (R)-3-hydroxybutyrate, ethyl (S)-3-hydroxybutyrate, ethyl 3-hydroxybutyrate, ethyl 3-hydroxyhexanoate, ethyl 2-methylbutyrate, ethyl isovalerate, butyl isovalerate, ethyl trans-2-butenoate, hexyl trans-2-butenoate, ethyl tiglate, methyl benzoate, ethyl benzoate, methyl salicylate, ethyl cinnamate, methyl jasmonate, isopentyl tiglate, dimethyl carbonate, dimethyl glutarate, diethyl succinate and diethyl phthalate. All except cis-vaccenyl acetate were assayed in group assays only.
* Model substrates for which direct UV/vis spectrophotometric assays exist.
• Value back calculated from full Michaelis-Menten kinetic data (Supplementary Fig. S1).

Supplementary Table S2. Compounds from Fig. 1 for which there is evidence of bioactivity in Drosophila.

Substrate	Nature of Evidence		References
	In vitro activity against known OR	Behavioural differences	
**Straight chain esters**			
Methyl myristate	✓	✓	[1]
Propyl acetate	✓	✓	[2]
Propyl propionate	✓		[2]
Propyl butyrate	✓		[3]
Propyl hexanoate	✓		[2]
Butyl acetate	✓	✓	[2]
Butyl butyrate	✓		[3]
Butyl pentanoate	✓		[2]
Pentyl acetate	✓	✓	[2]
Pentyl pentanoate	✓		[3]
Hexyl acetate	✓	✓	[2]
Hexyl butyrate	✓	✓	[2]
Hexyl hexanoate	✓	✓	[2]
**Cyclic alcohol moieties**			
Benzyl acetate	✓		[2]
Phenethyl acetate	✓	✓	[2]
**Branched and unsaturated alcohol moieties**			
Heptyl acetate	✓		[2]
Octyl acetate	✓		[2]
Nonyl acetate	✓		[2]
Decyl acetate	✓		[2]
Isobutyl acetate	✓		[2]
Isopentyl acetate	✓	✓	[2]
2-Methylbutyl acetate	✓		[2]
cis-3-Hexenyl acetate	✓		[2]
E2-Hexenyl acetate	✓	✓	[2]
cis-Vaccenyl acetate	✓	✓	[2]
Geranyl formate	✓		[2]
Geranyl acetate	✓		[2]
Neryl acetate	✓		[2]

Supplementary Table S3. Proteins closest in structure to EST6 using the Q-score calculated by the SALAMI server accessed on May 2016[4].

Protein Name	PDB Code	Seq ID (%)	Q Score
Manducta sexta JHE	2fj0A	22	0.9435
Lucilia cuprina αE7	4fnmA	26	0.9321
Drosophila melanogaster AChE	1qo9A	25	0.9001
Torpedo californica AChE	3m3dA	27	0.9000
Homo sapiens AChE	4m0eA	26	0.8985
Homo sapiens BChE	2j4cA	25	0.8967
Bos taurus Lipase	1aqlB	28	0.8961
Mus musculus AChE	2c0pA	26	0.8912
Ophiostoma piceae Esterase	4be4A	22	0.8861
Homo sapiens Lipase	1jmyA	29	0.8843

Supplementary Table S4. DOCKovalent[5] binding scores for the representative range of potential substrates docked into the active site of EST6.

Ester	Structure	Score*
Pentyl butyrate	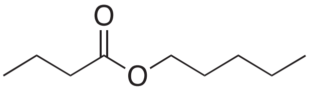	-24.8
Pentyl pentanoate	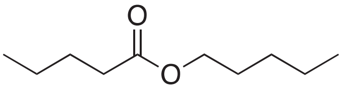	-6.04
Hexyl acetate	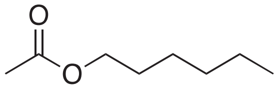	-29.1
Octyl acetate	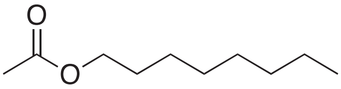	-22.0
Octyl propionate	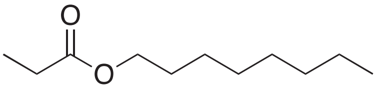	-22.4
Geranyl acetate	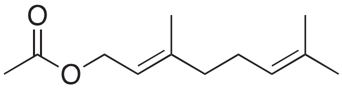	-18.3
Phenyl acetate	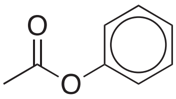	0.63
Phenethyl acetate	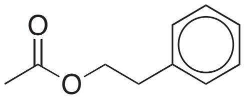	-26.5
cis-Vaccenyl acetate	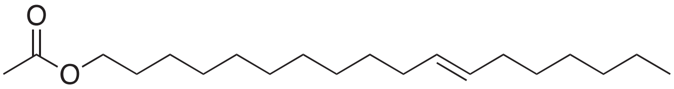	NA[†]

* Total score is calculated according to the physics-based scoring function implemented in DOCK3.7. The scoring function evaluates the ligand-protein electrostatic and van der Waals interactions and corrects for desolvation[5].
[†] No suitable binding poses (without steric clashes) obtained.

Supplementary Table S5. Data collection and refinement statistics for EST6-1. The diffraction data were indexed, integrated and scaled using Aimless and XDS[6,7]. Molecular replacement was used to obtain phases using the structure of LcαE7 with MOLREP[8]. Model building was carried out via iterative cycles of automated model building with BUCCANEER[9] and manual rebuilding with COOT[10]. Refinement was undertaken using REFMAC V 5.7.0032 accessed through the CCP4 suite of programs[11,12] and phenix.refine[13].

	**EST6-1**
**Data collection**	
Space group	$P2_12_12_1$
Cell dimensions	
a, *b*, *c* (Å)	67.7, 80.6, 107
α, β, γ (°)	90, 90, 90
Resolution (Å)	40.32-2.15 (2.22-2.15)
R_{sym} or R_{merge}	0.114 (0.331)
$CC_{1/2}$	0.996
CC*	0.995
I / σ /*I*	18.1 (4.51)
Completeness (%)	96.6 (73.4)
Redundancy	11.7 (10.4)
**Refinement**	
Resolution (Å)	40.32-2.15 (2.52-2.15)
No. reflections	31400
R_{work} / R_{free}	0.1554/0.2104 (0.1698/0.2325)
No. atoms	
Protein	520
Ligand/ion	1
Water	353
B-factors	
Protein	19.44
Ligand/ion	25.13
Water	22.11
R.m.s. deviations	
Bond lengths (Å)	0.008
Bond angles (°)	0.95
PDB ID	5THM

*Values in parentheses are for highest-resolution shell.

Supplementary Text

Supplementary Results

In order to corroborate the results of the immunohistochemical experiment showing different localization of EST6 and LUSH within the antennae we performed a set of male-male courtship experiments on *Est6* knock-down flies bearing RNAi constructs driven by *Est6* or *lush* Gal4 lines to demonstrate that *Est6* is not co-expressed with *lush*. Previous work has shown that *Est6*° null mutant flies exhibit lower levels of male-male courtship[14]. As expected, we observed a significant reduction in the male-male courtship index (CI) when the *UAS-RNAi* transgene was driven by $Est6^{Gal4}$ (12.20 ± 3.27% for $Est6^{Gal4}$/*UAS-RNAi Est6*), whereas the CI when the *UAS-RNAi* transgene was driven by $lush^{Gal4}$ (29.29 ± 4.48 for $lush^{Gal4}$/*UAS-RNAi Est6*) was not significantly different from the various parental genotypes (27.24 ± 4.88 for $lush^{Gal4}$, *25.60 ± 4.75 for* $Est6^{Gal4}$ and 23.00 ± 4.76 for *UAS-RNAi Est6)*. This result strongly suggests that EST6 and LUSH are not produced by the same cells of the sensilla since even an overlap of cells expressing both proteins would be expected to result in an intermediate phenotype.

Supplementary Methods

Flies. Additional lines used are *UAS-RNAi Est6* flies from the NIG Stock Center (Japan) and transgenic $Est6^{Gal4}$ flies described in Chertemps *et al.*[14]. Both stocks were maintained as described in the main text.

Courtship assays. Male-male courtship assays were performed as described in Chertemps *et al.*[14]. Briefly, all experiments were done under dim red lights at 25 ˚C (50% to 60% relative humidity). A single male (5 to 7 days old) was placed in a test chamber (30 mm diameter, 5 mm height) for 10 minutes before introducing a decapitated *CS* 'target' male. Courtship behaviour was observed over 10 minutes and a courtship index (CI) was calculated as the fraction of time spent in courtship activity over the observation period. The CIs of the parental control strains $lush^{Gal4}$, $Est6^{Gal4}$ and *UAS-RNAi Est6* were compared to the CIs of the $lush^{Gal4}$/*UAS-RNAi Est6* and $Est6^{Gal4}$/*UAS-RNAi Est6* strains, in which *Est6* inhibition is driven by either the *lush* or *Est6* promoters. At least 22 flies of each genotype were tested.

Supplementary References

1 Dweck, H. *et al.* Pheromones mediating copulation and attraction in Drosophila. *Proc. Natl. Acad. Sci. U. S. A.* **112**, doi:10.1073/pnas.1504527112 (2015).

2 Dweck, H. *et al.* Olfactory Preference for Egg Laying on Citrus Substrates in Drosophila. *Curr. Biol.* **23**, doi:10.1016/j.cub.2013.10.047 (2013).

3 Galizia, C., Münch, D., Strauch, M., Nissler, A. & Ma, S. Integrating heterogeneous odor response data into a common response model: A DoOR to the complete olfactome. *Chem. Senses* **35**, doi:10.1093/chemse/bjq042 (2010).

4 Margraf, T., Schenk, G. & Torda, A. The SALAMI protein structure search server. *Nucleic Acids Res.* **37**, doi:10.1093/nar/gkp431 (2009).

5 London, N. *et al.* Covalent docking of large libraries for the discovery of chemical probes. *Nat. Chem. Biol.* **10**, 1066-1072, doi:10.1038/nchembio.1666 (2014).

6 Evans, P. Scaling and assessment of data quality. *Acta Crystallogr. D Biol. Crystallogr.* **62**, doi:10.1107/S0907444905036693 (2006).

7 Kabsch, W. XDS. *Acta Crystallogr. D Biol. Crystallogr.* **66**, doi:10.1107/S0907444909047337 (2010).

8 Vagin, A. & Teplyakov, A. MOLREP: an Automated Program for Molecular Replacement. *J. Appl. Cryst.* **30**, doi:10.1107/S0021889897006766 (1997).

9 Cowtan, K. The Buccaneer software for automated model building. 1. Tracing protein chains. *Acta Crystallogr. D Biol. Crystallogr.* **62**, doi:10.1107/S0907444906022116 (2006).

10 Emsley, P. & Cowtan, K. Coot: model-building tools for molecular graphics. *Acta Crystallogr. D Biol. Crystallogr.* **60**, 2126-2132, doi:10.1107/S0907444904019158 (2004).

11 Murshudov, G. N. *et al.* REFMAC5 for the refinement of macromolecular crystal structures. *Acta Crystallogr. D Biol. Crystallogr.* **67**, doi:10.1107/S0907444911001314 (2011).

12 Winn, M. D. *et al.* Overview of the CCP4 suite and current developments. *Acta Crystallogr. D Biol. Crystallogr.* **67**, 235-242, doi:10.1107/s0907444910045749 (2011).

13 Afonine, P. V. *et al.* Towards automated crystallographic structure refinement with phenix.refine. *Acta Crystallogr. D Biol. Crystallogr.* **68**, 352-367, doi:10.1107/s0907444912001308 (2012).

14 Chertemps, T. *et al.* A carboxylesterase, Esterase-6, modulates sensory physiological and behavioral response dynamics to pheromone in Drosophila. *BMC Biol.* **10**, doi:10.1186/1741-7007-10-56 (2012).
